# Heteroatom Effects on Quantum Interference in Molecular Junctions: Modulating Antiresonances by Molecular Design

**DOI:** 10.1021/acs.jpcc.1c04242

**Published:** 2021-08-02

**Authors:** Luke J. O’Driscoll, Sara Sangtarash, Wei Xu, Abdalghani Daaoub, Wenjing Hong, Hatef Sadeghi, Martin R. Bryce

**Affiliations:** † Department of Chemistry, Durham University, Lower Mountjoy, Stockton Road, Durham DH1 3LE, U.K.; ‡ School of Engineering, University of Warwick, Coventry CV4 7AL, U.K.; § State Key Laboratory of Physical Chemistry of Solid Surfaces, iChEM, NEL, College of Chemistry and Chemical Engineering, Xiamen University, Xiamen 361005, China

## Abstract

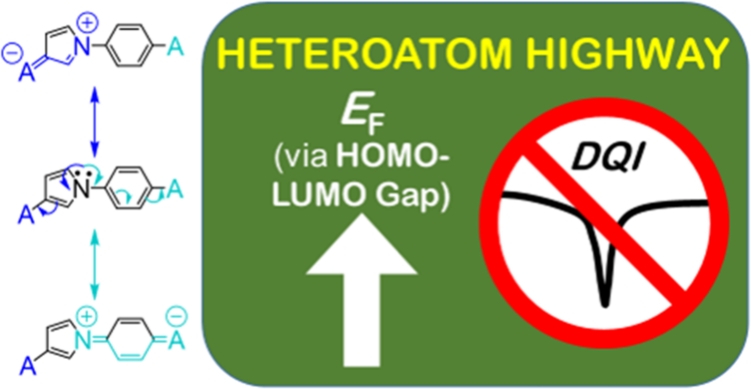

Controlling charge transport through molecular wires by utilizing quantum interference (QI) is a growing topic in single-molecular electronics. In this article, scanning tunneling microscopy-break junction techniques and density functional theory calculations are employed to investigate the single-molecule conductance properties of four molecules that have been specifically designed to test extended curly arrow rules (ECARs) for predicting QI in molecular junctions. Specifically, for two new isomeric 1-phenylpyrrole derivatives, the conductance pathway between the gold electrodes must pass through a nitrogen atom: this novel feature is designed to maximize the influence of the heteroatom on conductance properties and has not been the subject of prior investigations of QI. It is shown, experimentally and computationally, that the presence of a nitrogen atom in the conductance pathway increases the effect of changing the position of the anchoring group on the phenyl ring from *para* to *meta*, in comparison with biphenyl analogues. This effect is explained in terms of destructive QI (DQI) for the *meta*-connected pyrrole and shifted DQI for the *para*-connected isomer. These results demonstrate modulation of antiresonances by molecular design and verify the validity of ECARs as a simple “pen-and-paper” method for predicting QI behavior. The principles offer new fundamental insights into structure–property relationships in molecular junctions and can now be exploited in a range of different heterocycles for molecular electronic applications, such as switches based on external gating, or in thermoelectric devices.

## Introduction

Single-molecule conductance values have been determined for a diverse array of molecular wires trapped between metal electrodes since the development of specialized measurement techniques in the late 1990s and early 2000s.^1−3^ These methods include mechanically controlled break junction^4^ and scanning tunneling microscopy-break junction (STM-BJ)^5^ experiments. By combining these techniques with the power of organic synthesis, it has been widely demonstrated that substantial variation in the conductance of molecular wires can be achieved by small structural modifications, such as structural isomerism and/or the presence of heteroatoms.^6−13^ Particularly, in the case of π-conjugated systems, much of this behavior can be attributed to quantum interference (QI) effects,^14−16^ which are readily visualized in transmission functions derived from charge-transport simulations.^2,17−20^

The transmission function *T*(*E*) of a molecular junction is a plot of the probability of electrons with energy *E* passing from one electrode to the other through the molecule and is proportional to molecular conductance. *E* is usually considered relative to the system’s Fermi energy, *E*_F_. Calculated transmission functions from first-principles simulations reliably show qualitative agreement with experimental conductance studies.^2^ Quantitative agreement is more challenging due to difficulties such as the accurate determination of *E*_F_ using density functional theory (DFT).^17,21^ Sharp resonances coincident with the energies of molecular orbitals (e.g. the highest occupied and lowest unoccupied molecular orbitals, HOMO and LUMO, respectively) are key features of a typical transmission function. In low-bias conductance studies, *E*_F_ usually lies near the center of the HOMO–LUMO gap. Furthermore, the low-bias QI behavior of a molecular junction relates to the characteristics of the transmission function in the HOMO–LUMO gap. QI can be constructive (CQI) or destructive (DQI). Where CQI occurs, a smooth, featureless transmission curve is usually seen between the HOMO and LUMO resonances. A characteristic feature of DQI is a sharp antiresonance in the transmission curve where *T*(*E*) approaches zero.

This work considers two subcategories of DQI, based on the energy at which an antiresonance appears in the transmission function of a molecular junction. DQI refers to cases where an antiresonance occurs close to *E*_F_, and significantly reduced low-bias conductance would be expected relative to a similar system without an antiresonance. Shifted DQI (SDQI) refers to systems where an antiresonance occurs in the transmission function but does not lie close to *E*_F_ and so the conductance of the junction remains high in the low-bias regime.^19,20^ Where SDQI occurs, an antiresonance can even be shifted beyond the HOMO–LUMO gap,^20^ meaning that SDQI is not always readily distinguishable from CQI.

In addition to computationally demanding charge-transport simulations, many simpler methods exist to predict and rationalize the QI behavior of molecular wires. Some methods are based on structural considerations alone, such as “curly arrow” rules (CARs)^22−24^ and graphical or topological methods.^15,25,26^ Other methods require a mathematical or computational input, such as orbital symmetry approaches,^27,28^ QI maps,^29^ and magic ratio rules.^18,30−33^ These more straightforward methods necessarily have limitations to their scope compared to charge-transport simulations. They generally work well for bipartite hydrocarbon lattices but can be less accurate for molecular wires that incorporate more elaborate structural features, such as (i) deviation from a framework of fused six-membered rings; (ii) the inclusion of heteroatoms, either as substituents or within the lattice; or (iii) cross-conjugation.

Two of the present authors recently presented an extension to predictive CARs for QI behavior [extended curly arrow rules (ECARs)].^24^ This was in part inspired by work from another two of the present authors which showed that simple CARs as widely applied in molecular electronics^22^ “broke down” when applied to cross-conjugated anthraquinone derivatives.^8^ ECARs are a “pen-and-paper” method that can predict whether a given molecule will exhibit CQI, DQI, or SDQI. ECARs account for previously reported QI behavior of molecular wires containing heteroatoms, nonbipartite structures, and cross-conjugation.^24^ However, ECARs cannot predict the relative conductance of wires with respect to one another. Despite this, the conductance of structurally similar materials would usually be expected to follow the trend CQI ≥ SDQI > DQI. The rules^24^ are as follows:

### ECAR-1

Identify the two anchoring units of a molecular wire and replace one with a donor group (D) and the other with an acceptor group (A). If the D lone pair can be delocalized onto A using curly arrows, CQI is expected; if not, DQI is expected.

### ECAR-2

If DQI is expected, identify any electron-withdrawing groups (EWGs) or electron-donating groups (EDGs) present in the molecular wire. If EWGs are present, replace each anchor with D. If a lone pair from each D can be independently delocalized to the same EWG, SDQI is expected. If EDGs are present, replace each contact with A. If a lone pair (or negative charge) from the same EDG can be independently delocalized to each A, SDQI is expected. Otherwise, DQI is expected around *E*_F_.

To further test the validity of ECARs, we have designed and synthesized new heteroatom-containing molecular wires that differ structurally from those considered in the development of the rules. Specifically, the novel feature of these molecules is that when they are held between gold electrodes, the conductance pathway through these molecules must pass through a nitrogen atom. In contrast, a pathway comprising only carbon atoms existed in all of the examples used in the conception of ECARs.^24^ To our knowledge, this is the first study of QI effects in organic molecules where an all-carbon conductance pathway is not available between the anchoring groups. The predictions made by ECARs for these new wires have been tested experimentally using the STM-BJ technique and investigated computationally by calculating transmission functions using a simple tight-binding method and DFT-based material-specific charge-transport simulations.

## Methods

Full details of the synthesis and characterization of molecules **1–4** are given in the Supporting Information. In brief, the 1-phenylpyrrole derivatives **1** and **2** were prepared from 3-bromo-1-(triisopropylsilyl)pyrrole. The thiomethyl anchor was first installed through lithiation followed by treatment with dimethyl disulfide.^34^ The TIPS protecting group was then removed before forming the aryl–aryl *C*–*N* bond via Ullmann coupling^35^ with the appropriate bromothioanisole. The biphenyl species **3** and **4** were prepared based on a reported synthesis of **4**(36) using a Suzuki cross-coupling reaction between 3-(methylthio)phenylboronic acid and the appropriate bromothioanisole.

Molecular conductance measurements were performed using the lab-built STM-BJ technique, which has been reported in previous publications.^5,37^ In brief, molecular junctions were repeatedly formed by driving the gold tip in and out of contact with a gold substrate. Conductance was measured as a function of the gold tip-substrate displacement, which is mainly controlled by a piezo stack during the repeated formation of junctions (see Supporting Information for more details). All experiments were carried out in a solution of the target molecules (0.1 mM) in mesitylene under ambient conditions with a 0.1 V bias voltage. Logarithmically binned one-dimensional (1D) conductance histograms and two-dimensional (2D) conductance-displacement histograms were plotted by compiling at least 2000 molecular conductance-displacement traces. Statistical analysis was performed using the methods we reported previously.^37^

The molecular conductance behavior of molecules **1–4** was investigated computationally using DFT combined with quantum transport calculations.^21^ From the optimized geometry of each molecule in the gas phase and between two gold electrodes, we obtained a ground-state Hamiltonian from the Siesta^38^ implementation of DFT and combined it with the Gollum^21,39^ transport code to obtain a transmission coefficient *T*(*E*) for electrons with energy *E* passing from one electrode to the other (see Computational Methods in the Supporting Information for further details). The low-bias electrical conductance was then calculated from the Landauer formula *G* = *G*_0_*T*(*E*_F_), where *G*_0_ is the conductance quantum and *E*_F_ is the Fermi energy of the electrodes. The room temperature electrical conductance was obtained from the thermal averaging of *T*(*E*) (see Computational Methods in the Supporting Information).

## Results and Discussion

### Molecular Design

**Figure 1:**
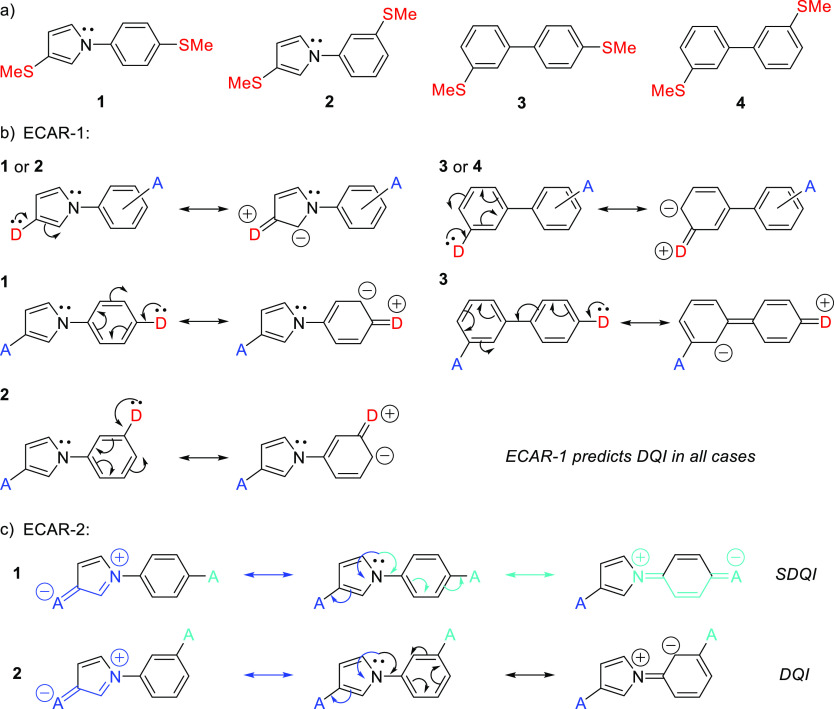
(a) Structures of the studied 1-phenylpyrrole (**1** and **2**) and biphenyl (**3** and **4**) wires; (b) application of ECAR-1 to the four wires—note that the choice of which anchor is replaced with D and which with A has no impact on the result of ECAR-1 and that it is not possible to delocalize a D lone pair onto the pyrrole nitrogen as no vacant orbitals are available; (c) application of ECAR-2 to wires **1** and **2**, for which the nitrogen lone pair can be used as an EDG. Different colored curly arrows represent different delocalization pathways indicated by correspondingly colored resonance arrows.

We set out to design molecules that could be used to test the validity of ECARs via investigation of their conductance behavior, both computationally and in break junction studies. To test the breadth of applicability of ECARs, we targeted molecules with a clear structural difference to those used in prior studies of QI. The isomeric 1-phenylpyrrole (i.e. *N*-phenylpyrrole) derivatives **1** and **2** (Figure 1a) contain a nitrogen atom that lies directly in the conductance pathway of the molecules, with no alternative through-bond route between the anchoring groups by which the nitrogen can be avoided. This is in contrast to the species studied previously to which ECARs were applied^24^ and should maximize the influence of the heteroatom on conductance properties. Past studies of QI effects in molecular junctions have only considered molecular backbones where a heteroatom-containing pathway exists in parallel to an all-carbon pathway^10−13^ or organometallic systems.^40,41^ Studies of molecular junctions where the conductance pathway *must* pass through one or more heteroatoms in the molecular backbone have been reported,^35,42^ for example, using oligophenyleneimines.^43^ However, to our knowledge, QI effects have not been investigated in such systems.

Each 1-phenylpyrrole isomer has a thiomethyl anchoring group in the pyrrole 3-position and a second thiomethyl anchor on the benzene ring, either *para* (**1**) or *meta* (**2**) to the pyrrole ring. As shown in Figure 1b, when applying ECAR-1, it is not possible to delocalize electrons from a D group at either anchoring position to an A group at the other for either isomer. However, the nitrogen lone pair can be used as an EDG for ECAR-2 (Figure 1c). For **1**, it is possible to independently delocalize the nitrogen lone pair to an A group in either anchoring position, so ECARs predict SDQI. For **2**, it is only possible to delocalize the nitrogen lone pair to an A group in the pyrrole-anchoring position (the anchoring group on the benzene ring is *meta* to the EDG, so delocalization is not possible); therefore, DQI is expected.

For comparison, the analogous biphenyl derivatives **3** and **4** (Figure 1a), in which the pyrrole ring is replaced by a benzene ring with the anchor in the *meta*-position, were investigated. Similar to **1** and **2**, this means that when applying ECAR-1, it is not possible to delocalize electrons from a D group at either anchoring position to an A group at the other position for either **3** or **4** (Figure 1b). As no EDGs or EWGs are present in **3** or **4**, ECAR-2 is not applicable and DQI is expected for both systems. As the four biaryl systems **1–4** form relatively short molecular wires, it was expected that their molecular conductance would be sufficiently high to measure experimentally despite the expected occurrence of DQI in three of the systems. The thiomethyl anchoring groups were selected for their proven and effective anchoring properties^44−47^ and good compatibility with the synthetic route.

It was anticipated that direct comparison between the 1-phenylpyrrole (**1** and **2**) and biphenyl (**3** and **4**) species could be complicated by differences in the torsional angle (θ) between the connected rings. The angle θ was not expected to vary significantly within each isomer pair, as the steric environment around the aryl–aryl bond remains the same. We, therefore, reasoned that any influence of θ would be overshadowed by comparing the relative effect of changing the position of the second anchoring group (i.e. that on the right of the structures in Figure 1a) from *para* to *meta* for the two isomeric pairs. If the prediction of ECARs is correct and **1** shows SDQI (and therefore higher low-bias conductance), while the other three species show DQI, then, the following relationship between molecular conductances *G*_*X*_ (where *X* is the molecule number) should hold around *E*_F_:
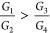
This means that a larger decrease in conductance is expected for the 1-phenylpyrrole backbone than the biphenyl backbone as the second anchor is changed from the *para*- to *meta*-position. In practice, the DFT-minimized conformations of **1–4** (see below, and Figure S13 and Table S1 in the Supporting Information) showed that θ was similar for all four species in the gas phase. In the DFT-minimized molecular junction conformations, θ differed by 6° in the 1-phenylpyrrole isomer pair and 8° in the biphenyl pair. We emphasize that these values relate only to the energy-minimized junction conformation. Experimental conductance measurements sample a broad range of conformations, where θ is likely to vary similarly for a given isomer pair. As the barrier to rotation is low at room temperature, we, therefore, anticipated that the proposed conductance relationship would remain valid.

### Molecular Conductance Studies

**Figure 2:**
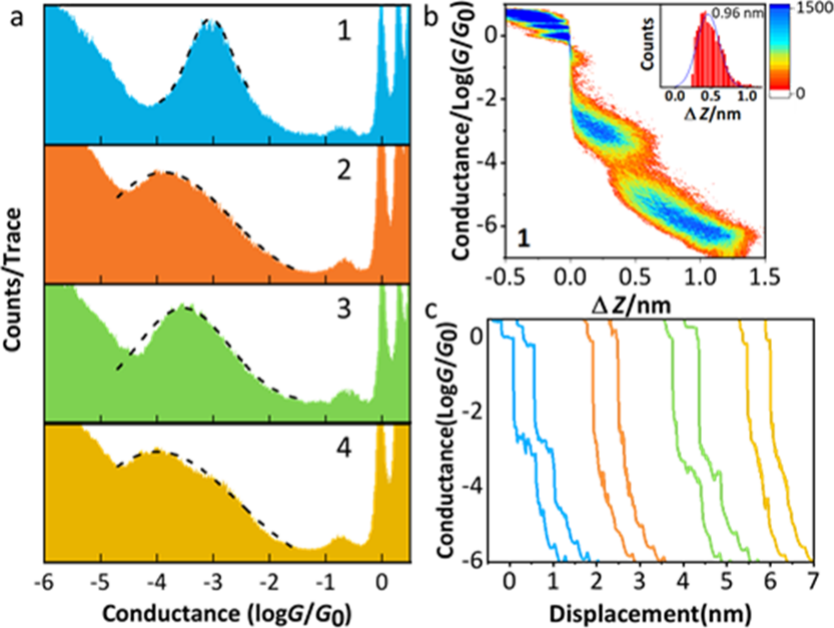
(a) Logarithmically binned conductance histograms of molecules **1–4**; (b) 2D conductance-displacement histogram of molecule **1** under 0.1 V bias voltage (2D histograms of molecules **2–4** are shown in Figure S11 in the Supporting Information), inset: length distribution; (c) representative conductance traces measured for molecules **1–4** (trace colors match those used in panel a).

The STM-BJ technique^5,37^ was used to investigate the molecular conductance of the four molecules (see Methods and the Supporting Information). 1D conductance histograms are shown in Figure 2a, with 2D histograms in Figures 2b and S11 in the Supporting Information and example conductance traces in Figure 2c. The most probable molecular conductances for the four molecules follow the trend **1** (10^–3.05^*G*_0_) > **3** (10^–3.50^*G*_0_) > **2** (10^–3.85^*G*_0_) > **4** (10^–4.05^*G*_0_). The broader conductance histograms observed for **2** and **4** (and to a lesser extent **3**) in comparison to **1** can be related to a wider range of possible junction configurations.^45^ A small shoulder is visible in the 1D histogram of **4** (Figure 2a). This minor feature may be caused by Au-π, rather than Au–S electronic coupling of a *meta*-anchored phenyl ring.^48^ However, previous examples of such behavior were observed only when the other anchor was *para*-connected, and a similar feature is not observed for **2** or **3**. The small peaks visible between 10^–1^ and 10^0^*G*_0_ in Figure 2a are attributed to the conductance of single molecules of mesitylene, which was used as the solvent in the measurements.^49^

The hypothesized relationship between the molecular conductances based on ECARs holds: the ratio between the most probable conductances of molecules **1** and **2** is 10^0.80^ (ca. 6.3), whereas that between **3** and **4** is only 10^0.55^ (ca. 3.5). Changing the position of the second anchoring group from *para* to *meta* has nearly double the effect for the 1-phenylpyrrole species **1** and **2** than it has for the biphenyl wires **3** and **4**. The higher conductance of **1** relative to **3** (and **2** relative to **4**) indicates that the 1-substituted 3-(methylthio)pyrrole unit affords improved conductance relative to a *meta*-linked benzene ring. Indeed, the conductance of **1** is comparable to that of the *para*-linked biphenyl species 4,4′-bis(methylsulfide)biphenyl,^37^**5**, which was determined to be 10^–3.10^*G*_0_ under the same experimental conditions used for **1–4** (Figure S10).

A similar trend is observed if the relative conductances of molecules **2**, **3**, and **4** are compared. Each has a *meta*-anchored benzene bound to a second aromatic system, respectively, 3-(methylthio)pyrrole (via the pyrrole 1-position), *para-*(methylthio)benzene, or *meta*-(methylthio)benzene. As molecular conductance increases in the sequence **4** < **2** < **3**, 1-linked 3-(methylthio)pyrrole can be considered an intermediate between *para*- and *meta*-(methylthio)benzene. This trend in relative conductance is notably compatible with the QI behavior that ECARs predict for wires comprising only a *meta*-benzene (DQI), 1,3-difunctionalised pyrrole (SDQI), or *para*-benzene (CQI).

### Charge-Transport Simulations

**Figure 3:**
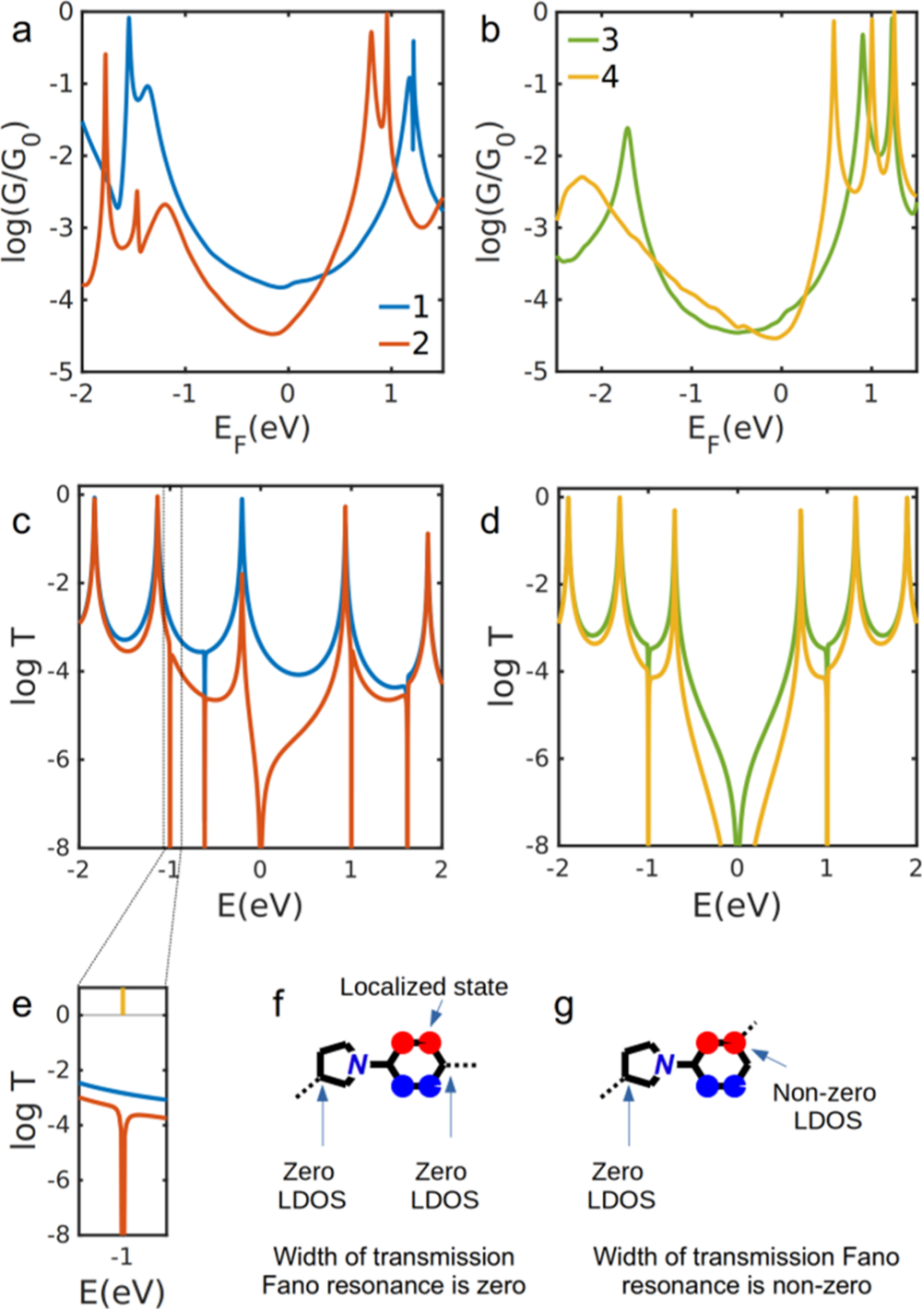
Calculated electron transport through molecules **1–4** between gold electrodes using DFT material-specific Hamiltonians (a,b) and a tight-binding model (c,d). For the tight-binding model, site energies are 0 for all atoms except the nitrogen atom, which has a site energy of −0.5. All couplings between connected sites are −1. Expansion of the indicated region of panel c (e), showing *T*(*E*) for **1** and **2** around *E* = −1 eV, and the energy level coincident with this energy range. Tight-binding molecular orbitals for the energy level around *E* = −1 eV, showing that the LDOS is zero at both connection points of **1** (f) but non-zero at one connection point of **2** (g), resulting in the respective absence or presence of a Fano resonance around this energy in panel (e).

Figure 3a,b shows the calculated conductance for molecules **1–4** between gold electrodes based on DFT material-specific Hamiltonians. The conductance of **1** is higher than **2** for a wide range of *E*_F_ around the DFT Fermi energy (*E*_F_ = 0 eV), and the conductance of **3** is higher than that of **4** around *E*_F_ = 0 eV. This is in qualitative agreement with the experimentally determined conductance values (Figure 2), as is the trend in molecular conductance at *E*_F_ = 0 eV (see Table S2 in the Supporting Information), which decreases in the sequence **1** > **3** > **2** > **4**. (The possibility that the relative conductance was significantly influenced by different anchoring geometries^50^ of different isomers was ruled out as described in the Supporting Information and Figure S15). Furthermore, Figure 3a,b shows that for *E*_F_ < ca. 0.25 eV, *G*_1_ > *G*_2_ and *G*_3_ is similar to or < *G*_4_. This agrees with the ECARs-predicted relationship between the four molecular conductances. Taking the values at *E*_F_ = 0 eV as an example, the ratio of the conductances of **1** and **2** is 10^0.57^ (ca. 3.7) and that between **3** and **4** is 10^0.24^ (ca. 1.7). Similar to the trend observed in the STM-BJ data, the effect of switching the second anchoring group from *para* to *meta* for the 1-phenylpyrrole species is around twice as large as for the biphenyl wires. However, the DQI-mediated antiresonance feature near *E*_F_ that was predicted using ECARs is not clearly visible in the transmission function of **2**, **3**, or **4**. We attribute this to the effect of σ-orbitals on transport.^51^ Molecules **1–4** are short, and therefore the contribution to transport from σ-orbitals is higher than that from π-orbitals at energies around the antiresonance feature, causing it to be masked.

To illustrate this effect, we note that transmission functions calculated using a simple tight-binding method with a single π-orbital per atom (Figure 3c,d) show clear antiresonance features in the HOMO–LUMO gap for **2**, **3**, and **4** but not for **1**. Note that additional sharp features can be seen outside the HOMO–LUMO gap, at *E* ≈ ± 1 eV for **2–4** and *E* ≈ −0.6 and +1.6 eV for **1** and **2**. The origin of these features was investigated by calculating the tight-binding energy levels and corresponding wavefunctions for the 1-phenylpyrrole molecular core of **1** and **2** (Figure S14). A transmission resonance is usually expected close to the energy levels of a molecule in a junction. As exemplified in Figure 3e, although an energy level exists at *E* = −1 eV for the molecular core (indicated by the orange line in Figure 3e), no associated resonance is observed for **1** in the tight-binding transmission function, whereas for **2**, a very narrow resonance can be seen. This can be understood by examination of the wavefunctions of the molecular core (Figures 3f,g and S14 in the Supporting Information). The width of a transmission resonance is proportional to the sum of the density of states [local density of state (LDOS), i.e., modulus squared of wavefunctions] at the connection points to electrodes.^21^ When both LDOSs are zero, a resonance with zero width (a “vanishing resonance”) is expected, as seen for **1** in Figure 3f. In contrast, when only one LDOS is non-zero, as seen for **2** in Figure 3g, a resonance or Fano resonance is expected. A Fano resonance is normally due to a localized state (e.g. that shown in Figure 3f,g) that interacts weakly with continuum states. The feature observed for **2** close to *E* = −1 eV is, therefore, a Fano resonance (a resonance attached to an antiresonance), but the amplitude of the resonance is small because of a large asymmetry in the self-energies due to the coupling to the left and right electrodes.

To further demonstrate that the absence of antiresonance features in the DFT calculations is due to conduction through the σ-orbitals,^51^ the electrical conductance of extended analogues of molecules **1** and **2** (molecules **6** and **7** in Figure S12 in the Supporting Information) was calculated. Phenylacetylene units were added between the molecular core and the anchoring groups to lengthen the conductance pathway and weaken the effect of σ-channels on total transport. The resulting calculations (see Figure S12 of the Supporting Information) show that the antiresonance feature predicted by ECARs is present for **7** (i.e., it is no longer masked by σ-orbital contributions), whereas no antiresonance feature is observed in the HOMO–LUMO gap for **6**. The observed QI behavior agrees with the predictions of ECARs and the simple tight-binding study.

## Conclusions

Molecular wires **1** and **2**, based on 1-phenylpyrrole, were designed and synthesized to test recently reported ECARs for predicting QI behavior.^24^ By comparison with analogous biphenyl wires **3** and **4**, it was shown using STM-BJ studies that the presence of an unavoidable nitrogen atom in the through-bond conductance pathway increases the effect of changing the position of the anchoring group on the phenyl ring from *para* (**1**) to *meta* (**2**). This agrees with ECARs which predict DQI near *E*_F_ for **2–4** (i.e. a low low-bias conductance) and SDQI for **1** (i.e. a higher low-bias conductance due to a shifted antiresonance). The experimental results are supported by charge-transport simulations of the measured molecules and extended analogues. This work verifies the validity of ECARs as a “pen-and-paper” method for predicting QI behavior and will, therefore, have an impact on the design criteria of new molecular wires.

Despite the absence of an alternating pathway of single and double bonds between the anchoring units, the conductance of **1** is comparable to that of linearly conjugated 4,4′-bis(methylsulfide)biphenyl, **5**. 1,3-Disubstituted pyrroles therefore represent a prototype of heteroaromatic units that can be used to add SDQI behavior to a molecular wire without significantly reducing low-bias conductance. These results offer new fundamental understanding of structure–property relationships in molecular junctions which can now be exploited in a range of molecular electronic applications such as switches based on external gating or in thermoelectric devices.

## Supporting Information Available

Synthetic details and molecular characterization; general experimental and computational methods; ^1^H NMR spectra; ^13^C NMR spectra; synthetic procedures; UV-visible spectra; single-molecule conductance experiments; comparison of logarithmically binned conductance histograms; 2D conductance-displacement histograms; illustration of molecules; DFT-optimized configurations; torsion angle (θ) between aromatic rings; calculated molecular conductances; molecular orbitals; tight-binding orbitals and transmissions; and transmission coefficients (PDF)
