# Supplementary material for: Heteroatom Effects on Quantum Interference in Molecular Junctions: Modulating Antiresonances by Molecular Design
Source: J Phys Chem C Nanomater Interfaces. 2021 Aug 2;125(31):17385–91. doi: 10.1021/acs.jpcc.1c04242 (PMC8397347; doi:10.1021/acs.jpcc.1c04242)
Supplement: Supplementary file 1 — jp1c04242_si_001.pdf [file jp1c04242_si_001.pdf]

SUPPORTING INFORMATION

Heteroatom Effects on Quantum Interference in Molecular Junctions: Modulating Antiresonances by Molecular Design

Luke J. O'Driscoll,[†] Sara Sangtarash,[‡] Wei Xu,[§] Abdalghani Daaoub,[‡] Wenjing Hong,[§,*] Hatef Sadeghi[‡,*] and Martin R. Bryce[†,*]

[†] Department of Chemistry, Durham University, Lower Mountjoy, Stockton Road, Durham, DH1 3LE, UK

[‡] School of Engineering, University of Warwick, Coventry, CV4 7AL, UK

[§] State Key Laboratory of Physical Chemistry of Solid Surfaces, iChEM, NEL, College of Chemistry and Chemical Engineering, Xiamen University, Xiamen 361005, China

CONTENTS

**1**	**Synthesis and characterization**	**S2**
**1.1**	**General experimental methods**	**S2**
**1.2**	**Synthetic procedures**	**S2**
**1.3**	**^{1}H NMR spectra of compounds used in conductance studies**	**S7**
**1.4**	**^{13}C NMR spectra of compounds used in conductance studies**	**S11**
**1.5**	**UV-visible spectra of molecular wires 1-4**	**S15**
**2**	**Single molecule conductance experiments**	**S16**
**2.1**	**Experimental methods**	**S16**
**2.2**	**Supplementary experimental figures**	**S16**
**3**	**Computational studies**	**S18**
**3.1**	**Computational methods**	**S18**
**3.2**	**Supplementary computational figures, tables and discussion**	**S18**
**4**	**References**	**S22**

1. SYNTHESIS AND CHARACTERIZATION

1.1: General experimental methods

4,4′-bis(methylsulfide)biphenyl, **5**, was available in the Xiamen laboratory from a previous study.[1] Synthetic reagents were purchased commercially and used as received unless otherwise stated. Dimethyl disulfide was stored over molecular sieves. Anhydrous solvents were prepared using an Innovative Technology solvent purification system and stored in ampoules under argon, with the exception of anhydrous 1,4-dioxane (Acros Organics Extra Dry Acroseal®, used as received). Thin-layer chromatography (TLC) analysis was carried out using Merck silica gel 60 F_{254} TLC plates and spots were visualized using a UV lamp emitting at 365 or 254 nm. Column chromatography was performed using silica gel 60A (40-63 µm) purchased from Fluorochem. ^{1}H and ^{13}C NMR spectroscopy was carried out on a Bruker AV400 NMR spectrometer. For ^{1}H NMR spectra, chemical shifts are reported relative to the residual solvent peak (7.26 ppm for $CHCl_3$) and for ^{13}C NMR spectra, chemical shifts are reported relative to the solvent peak (77.16 ppm for $CDCl_3$). These, and any other residual solvent peaks were referenced to values reported in the literature.[2] All NMR spectra were processed using MestReNova V12. ESI mass spectrometry was carried out using a TQD mass spectrometer (Waters Ltd, UK) in flow injection analysis mode with acetonitrile as the mobile phase, subsequent accurate mass measurements used a QtoF Premier mass spectrometer (Waters Ltd, UK). ASAP mass spectrometry (including accurate mass measurements) was carried out using an LCT Premier XE mass spectrometer (Waters Ltd, UK) using TOF detection. Exact mass measurements were processed using Elemental Composition 4.0 embedded within MassLynx 4.1 (Waters Ltd, UK). Melting points were determined using a Stuart SMP40 machine with a ramping rate of 3 °C min^{-1}. The videos produced by the machine were analyzed manually to determine the melting point. Elemental analysis was performed on an Exeter Analytical E-440 machine.

Unless otherwise stated, reactions were conducted under an argon atmosphere. Where anhydrous solvents were used, glassware was first dried under vacuum using a heat gun, then filled directly with argon. Solvents and liquid reagents were added by syringe or cannula, and solid reagents were added under a positive pressure of argon. Degassing was conducted by bubbling argon through the reaction mixture using an argon-filled balloon fitted with a syringe needle. All mixed solvents in this work were prepared as v/v mixtures.

1.2: Synthetic procedures

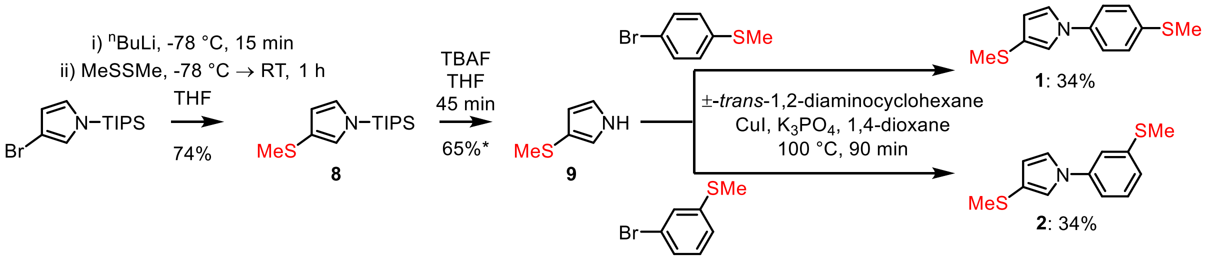

Scheme S1. Synthesis of 1-phenylpyrrole wires **1** and **2**. *Isolated yield: quantitative conversion was anticipated but separation of the product from the TIPSOH by-product required distillation and some mixed fractions were obtained. These were not fully purified owing to concerns over the long-term stability of the deprotected pyrrole.

3-methylthio-1-(triisopropylsilyl)pyrrole (8)

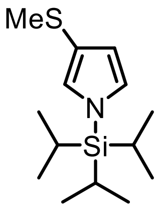

N.B. Unpleasant-smelling by-products, presumed to include methanethiol, form in this reaction. We recommend treating the aqueous layers and used glassware with bleach overnight to destroy these materials, and to ensure appropriate containment is used when handling the organic layers.

3-bromo-1-(triisopropylsilyl)pyrrole (3.30 mL, 12.7 mmol, 1 eq.) was dissolved in anhydrous THF (100 mL) in a dry flask under Ar. The mixture was degassed (Ar, 10 min) and cooled to −78 °C before ^{n}BuLi (2.0 M in hexanes, 14 mL, 28 mmol, 2.2 eq.) was added carefully over 10 min. The mixture was stirred at −78 °C for a further 15 min then dimethyl disulfide (2.5 mL, 28 mmol, 2.2 eq.) was added and the reaction was allowed to warm slowly to RT. After 1 h TLC indicated the reaction was complete, and it was quenched by careful addition of deionized H_2O (30 mL). The quenched mixture was degassed (Ar, 3 h), venting the exhaust gases through an oxidizing trap, to minimize the presence of foul-smelling volatile by-products prior to work-up. The mixture was diluted with additional deionized H_2O (70 mL) then extracted with CH_2Cl_2 (3 × 75 mL). The combined organic layers were washed with brine (2 × 75 mL) then dried ($MgSO_4$) before the solvent was removed *in vacuo* affording a yellow oil (3.96 g). The crude product was purified by column chromatography (5 cm Ø, 200 mL SiO_2, eluting with hexane until a higher-R_f impurity eluted[*], then gradient elution from hexane to 94:6 hexane/EtOAc) which afforded **8** as a pale yellow oil, which darkened upon standing under ambient conditions (2.54 g, 74%).

^{1}H NMR (400 MHz, $CDCl_3$) δ 6.76 (dd, *J* = 2.2, 1.4 Hz, 2H), 6.74 (dd, *J* = 2.7, 2.2 Hz, 1H), 6.32 (dd, *J* = 2.7, 1.4 Hz, 1H), 2.36 (s, 3H), 1.42 (hept, *J* = 7.5 Hz, 3H), 1.09 (d, *J* = 7.5 Hz, 18H).

^{13}C NMR (101 MHz, $CDCl_3$) δ 125.8, 125.1, 116.8, 113.6, 20.6, 17.9, 11.7.

MS (ESI-TOF): m/z: 270.2 $[M+H]^+$

HRMS (ESI-TOF): m/z $[M+H]^+$ calculated for $C_{14}H_{28}NSSi$, 270.1712; found, 270.1702

3-methylthiopyrrole (9)

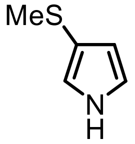

This reaction was not conducted under dry conditions or inert atmosphere.

8 (2.53 g, 9.39 mmol, 1 eq.) was dissolved in THF (50 mL) and stirred at RT. TBAF (1.0 M in THF, 10 mL, 10 mmol, 1.06 eq.) was added and the reaction was stirred at RT for 45 min, then quenched with saturated $NH_4Cl_{(aq)}$ (100 mL) and extracted with toluene (3 × 50 mL). The combined organic layers were washed with deionized water (3 × 50 mL) and brine (50 mL) then dried ($MgSO_4$) before removing the solvent *in vacuo* to afford a brown oil (2.46 g). NMR spectroscopy and mass spectrometry indicated that this was a mixture of **9** and TIPS-OH[†]. Using a kugelrohr, the TIPS-OH was removed by distillation (ca. 60 °C, ca. 0.3 mbar), leaving **9** of sufficient purity for the following reactions (< 5 mol % TIPS-OH remained based on ^{1}H NMR integrals) as a brown oil (690 mg, 65%[‡]). This material was used without further purification owing to concerns over the long-term stability of the *N*-unsubstituted pyrrole.

^{1}H NMR (400 MHz, $CDCl_3$) δ 8.23 (bs, 1H), 6.83 (apparent td, *J* = 2.1, 1.5 Hz, 1H), 6.78 (apparent td, *J* = 2.7, 2.1 Hz, 1H), 6.29 (apparent td, *J* = 2.7, 1.5 Hz, 1H), 2.36 (s, 3H).

^{13}C NMR (101 MHz, $CDCl_3$) δ 119.9, 118.9, 115.3, 112.0, 20.8.

MS (ESI-TOF): m/z: 114.0 $[M+H]^+$

HRMS (ESI-TOF): m/z $[M+H]^+$ calculated for C_5H_8NS, 114.0377; found, 114.0370

[*] Visible using a $KMnO_4$ TLC stain, R_f ca. 0.25 in hexane.

[†] Signals at 1.05 ppm in the ^{1}H NMR spectrum and at 17.8 and 12.4 ppm in the ^{13}C spectrum agree with reported values for TIPS-OH.[3]

[‡] Additional **9** contaminated with ca. 40 mol% TIPS-OH (416 mg) was collected in the final distilled fraction, but was not purified further or used in subsequent reactions.

3-methylthio-1-(4-methylthiophenyl)pyrrole (1)

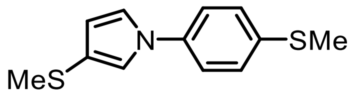

Based on previously reported conditions for C-N coupling of pyrrole derivatives and aryl halides.[4-5]

N.B. This compound has a slightly unpleasant smell.

9[§] (221 mg, 1.95 mmol, 1 eq.) and 4-bromothioanisole (793 mg, 3.90 mmol, 2 eq.) were dissolved in anhydrous 1,4-dioxane (20 mL) in a dry flask under Ar at RT and the solution was degassed (Ar, 20 min). CuI (372 mg, 1.95 mmol, 1 eq.), K_3PO_4 (1.24 g, 5.84 mmol, 3 eq.) and (±)-*trans*-1,2-diaminocyclohexane (0.47 mL, 3.9 mmol, 2 eq.) were added and the reaction was heated to 100 °C for 1 h, after which TLC indicated the reaction was complete. After cooling to RT the mixture was filtered through a pad of celite, washing with CH_2Cl_2 (ca. 150 mL) to ensure all soluble materials were collected. The filtrate was washed with 1 M $NaOH_{(aq)}$ (3 × 50 mL) and deionized H_2O (3 × 50 mL) then the organic phase was dried ($MgSO_4$) before the solvent was removed *in vacuo*, affording a brown oil (872 mg). The crude product was purified by column chromatography (2 cm Ø, 75 mL SiO_2, eluting with hexane to remove excess 4-bromothioanisole, then 99:1 hexane/EtOAc to elute the product) which afforded **1** as a cream colored solid (156 mg, 34%).

^{1}H NMR (400 MHz, $CDCl_3$) δ 7.33 – 7.26 (m, 4H), 7.05 (dd, *J* = 2.3, 1.7 Hz, 1H), 7.01 (dd, *J* = 2.9, 2.3 Hz, 1H), 6.36 (dd, *J* = 2.9, 1.7 Hz, 1H), 2.51 (s, 3H), 2.40 (s, 3H).

^{13}C NMR (101 MHz, $CDCl_3$) δ 137.9, 136.1, 128.1, 121.0, 120.6, 120.2, 117.8, 113.6, 20.3, 16.5.

MS (ASAP-TOF): m/z: 236.0 $[M+H]^+$, 235.1 $[M]^{+\cdot}$, 189.1 $[M+H-SMe]^{+\cdot}$

HRMS (ASAP-TOF): m/z $[M+H]^+$ calculated for $C_{12}H_{14}NS_2$, 236.0568; found, 236.0581

elem. anal.: Anal. Calcd for $C_{12}H_{13}NS_2$: C, 61.24; H, 5.57; N, 5.95. Found: C, 61.41; H, 5.61; N, 5.82.

m.p.: 46 – 47.5 °C

3-methylthio-1-(3-methylthiophenyl)pyrrole (2)

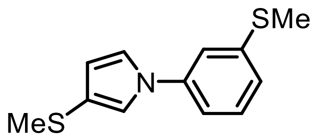

Based on previously reported conditions for C-N coupling of pyrrole derivatives and aryl halides[4-5]

N.B. This compound has a slightly unpleasant smell.

9[**] (334 mg, 2.95 mmol, 1 eq.) and 3-bromothioanisole (0.80 mL, 5.9 mmol, 2 eq.) were dissolved in anhydrous 1,4-dioxane (30 mL) in a dry flask under Ar at RT and the solution was degassed (Ar, 20 min). CuI (562 mg, 2.95 mmol, 1 eq.), K_3PO_4 (1.88 g, 8.86 mmol, 3 eq.) and (±)-*trans*-1,2-diaminocyclohexane (0.71 mL, 5.9 mmol, 2 eq.) were added and the reaction was heated to 100 °C for 1 h, after which TLC indicated the reaction was complete. After cooling to RT the mixture was filtered through a pad of celite, washing with CH_2Cl_2 (ca. 150 mL) to ensure all soluble materials were collected. The filtrate was washed with 1 M $NaOH_{(aq)}$ (3 × 50 mL) and

[§] As described above, but assumed to be pure for the basis of stoichiometric calculations.
[**] As described above, but assumed to be pure for the basis of stoichiometric calculations.

deionized H_2O (3 × 50 mL) then the organic phase was dried ($MgSO_4$) before the solvent was removed *in vacuo*, affording a brown oil (1.4 g). The crude product was purified by repeated column chromatography (1: 3 cm Ø, 150 mL SiO_2, gradient elution from hexane to 94:6 hexane/EtOAc; 2: 2 cm Ø, 75 mL SiO_2, eluting with hexane to remove excess 4-bromothioanisole, then 98:2 hexane/EtOAc to elute the product) which afforded **2** as a yellow oil which darkened over time even if stored at −3 °C in darkness (235 mg, 34%). NMR spectroscopy after a month of storage indicated that this darkening is not associated with any significant decomposition.

^{1}H NMR (400 MHz, $CDCl_3$) δ 7.33 (apparent t, *J* = 7.9 Hz, 1H), 7.22 (apparent t, *J* = 2.0 Hz, 1H), 7.15 – 7.09 (m, 2H), 7.07 (dd, *J* = 2.3, 1.7 Hz, 1H), 7.03 (dd, *J* = 3.0, 2.3 Hz, 1H), 6.37 (dd, *J* = 3.0, 1.7 Hz, 1H), 2.52 (s, 3H), 2.41 (s, 3H).

^{13}C NMR (101 MHz, $CDCl_3$) δ 140.8, 140.7, 130.0, 123.7, 120.6, 120.3, 118.2, 118.0, 117.1, 113.7, 20.3, 15.8.

MS (ASAP-TOF): m/z: 236.0 $[M+H]^+$, 235.1 $[M]^{+\cdot}$, 189.1 $[M+H\text{-}SMe]^{+\cdot}$

HRMS (ASAP-TOF): m/z $[M+H]^+$ calculated for $C_{12}H_{14}NS_2$, 236.0568; found, 236.0560

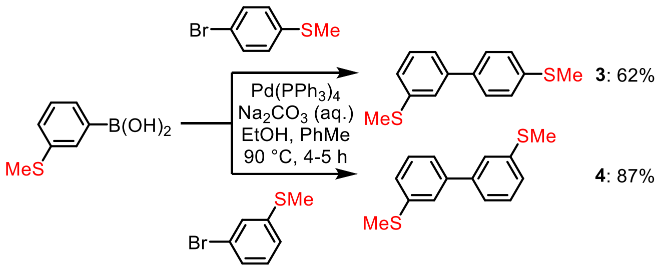

Scheme S2. Synthesis of biphenyl wires **3** and **4**.

3,4'-bis(methylthio)biphenyl (3)

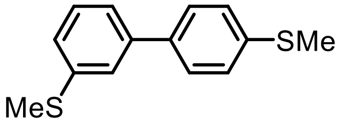

Based on a literature procedure,[6] with the modifications described for **4** below.

This reaction was not conducted under dry conditions, but care was taken to exclude oxygen.
3-(Methylthio)phenylboronic acid (375 mg, 2.23 mmol, 1.5 eq.), 4-bromothioanisole (302 mg, 1.49 mmol, 1 eq.), EtOH (4 mL), toluene (12 mL) and an aqueous solution of Na_2CO_3 (2 M, 3.35 mL, 6.7 mmol, 4.5 eq.) were added to a flask, stirred at RT and placed under Ar. The mixture was degassed (Ar, 45 min), $Pd(PPh_3)_4$ (86 mg, 0.075 mmol, 0.05 eq.) was added and the mixture was degassed for 5 more min, then heated to 90 °C. All solids suspended in the mixture had dissolved upon reaching this temperature. The reaction was stirred at 90 °C for 4 h, after which time TLC indicated the reaction was complete. The mixture was cooled to RT then diluted with 10 mL sat. $NaHCO_3$. The organic layer was separated, and the aqueous layer extracted with EtOAc (3 × 20 mL). The combined organic layers were dried ($MgSO_4$) before the solvent was removed *in vacuo* to afford a brown oil (576 mg). The crude product suspended on celite (10 mL, from CH_2Cl_2) then purified by column chromatography (3 cm Ø, 200 mL SiO_2, gradient elution from hexane to 19:1 hexane/EtOAc) which afforded a white solid. Remaining impurities were removed by recrystallization; the impure material was dissolved in a minimum of CH_2Cl_2 upon which hexane was layered. The solvent was allowed to slowly mix and evaporate, and after several days small white crystals formed in the concentrated solution. After filtration and drying in vacuo, **3** was obtained as a white solid (154 mg, 42%). A second crop of crystals was grown from the dried filtrate in the same manner, yielding further **3** as a white solid (72 mg). Total yield: 227 mg (62%).

^{1}H NMR (400 MHz, CD_2Cl_2) δ 7.55 – 7.51 (m, 2H), 7.47 – 7.44 (m, 1H), 7.38 – 7.34 (m, 2H), 7.34 – 7.30 (m, 2H), 7.25 – 7.20 (m, 1H), 2.53 (s, 3H), 2.52 (s, 3H).

^{13}C NMR (101 MHz, CD_2Cl_2) δ 141.6, 139.8, 138.8, 137.7, 129.7, 127.9, 127.1, 125.6, 125.1, 124.0, 16.1, 16.0.

MS (ASAP-TOF): m/z: 247.1 $[M+H]^+$, 246.1 $[M]^{+\cdot}$, 200.1 $[M+H\text{-}SMe]^{+\cdot}$

HRMS (ASAP-TOF): m/z $[M+H]^+$ calculated for $C_{14}H_{15}S_2$, 247.0615; found, 247.0599

elem. anal.: Anal. Calcd for $C_{14}H_{14}S_2$: C, 68.25; H, 5.73. Found: C, 68.55; H, 5.69.

m.p.: 54 – 55.5 °C

3,3'-bis(methylthio)biphenyl (4)

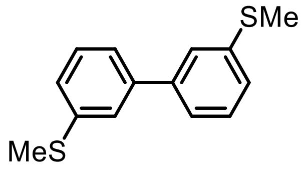

Based on a published procedure,[6] although a higher temperature than the reported 80 °C was required for the reaction to proceed.

This reaction was not conducted under dry conditions, but care was taken to exclude oxygen.
3-(Methylthio)phenylboronic acid (375 mg, 2.23 mmol, 1.5 eq.), 3-bromothioanisole (0.2 mL, 1.49 mmol, 1 eq.), EtOH (4 mL), toluene (12 mL) and an aqueous solution of Na_2CO_3 (2 M, 3.35 mL, 6.7 mmol, 4.5 eq.) were added to a flask, stirred at RT and placed under Ar. The mixture was degassed (Ar, 45 min), $Pd(PPh_3)_4$ (17 mg, 0.015 mmol, 0.01 eq.) was added and the mixture was degassed for 5 more min, then heated to 80 °C. All solids suspended in the mixture had dissolved upon reaching this temperature. The reaction was stirred at 80 °C for 18 h, after which time TLC indicated no reaction had occurred. Additional $Pd(PPh_3)_4$ (69 mg, 0.060 mmol, 0.04 eq.) was added and the temperature increased to 90 °C. After 5 h, TLC indicated the reaction was complete. The mixture was cooled to RT then diluted with sat. $NaHCO_3$ (10 mL). The organic layer was separated, and the aqueous layer extracted with EtOAc (3 × 10 mL). The combined organic layers were dried ($MgSO_4$) before the solvent was removed *in vacuo* to afford a brown solid (570 mg). The crude product was purified by column chromatography (3 cm Ø, 200 mL SiO_2, gradient elution from hexane to 9:1 hexane/EtOAc) which afforded an off-white solid. Remaining impurities were removed by recrystallization; the impure material was dissolved in a minimum of CH_2Cl_2 upon which hexane was layered. The solvent was allowed to slowly mix and evaporate, and after several days white needles formed in the concentrated solution. After filtration and drying in vacuo, **4** was obtained as white needles (248 mg). A second crop of crystals was grown from the dried filtrate in the same manner, yielding further **4** as a white solid (72 mg). Total yield: 320 mg (87%).

^{1}H NMR (400 MHz, CD_2Cl_2) δ 7.47 – 7.44 (m, 2H), 7.39 – 7.32 (m, 4H), 7.28 – 7.22 (m, 2H), 2.53 (s, 6H).

^{13}C NMR (101 MHz, CD_2Cl_2) δ 141.9, 139.9, 129.7, 125.9, 125.5, 124.4, 16.1.

MS (ASAP-TOF): m/z: 247.1 $[M+H]^+$, 246.1 $[M]^{+\cdot}$, 200.1 $[M+H\text{-}SMe]^{+\cdot}$

HRMS (ASAP-TOF): m/z $[M+H]^+$ calculated for $C_{14}H_{15}S_2$, 247.0615; found, 247.0636

elem. anal.: Anal. Calcd for $C_{14}H_{14}S_2$: C, 68.25; H, 5.73. Found: C, 68.38; H, 5.68.

m.p.: 55 – 56 °C (lit.[7] 54.5 – 55.5 °C)

1.3: ^{1}H NMR spectra of compounds used in conductance studies

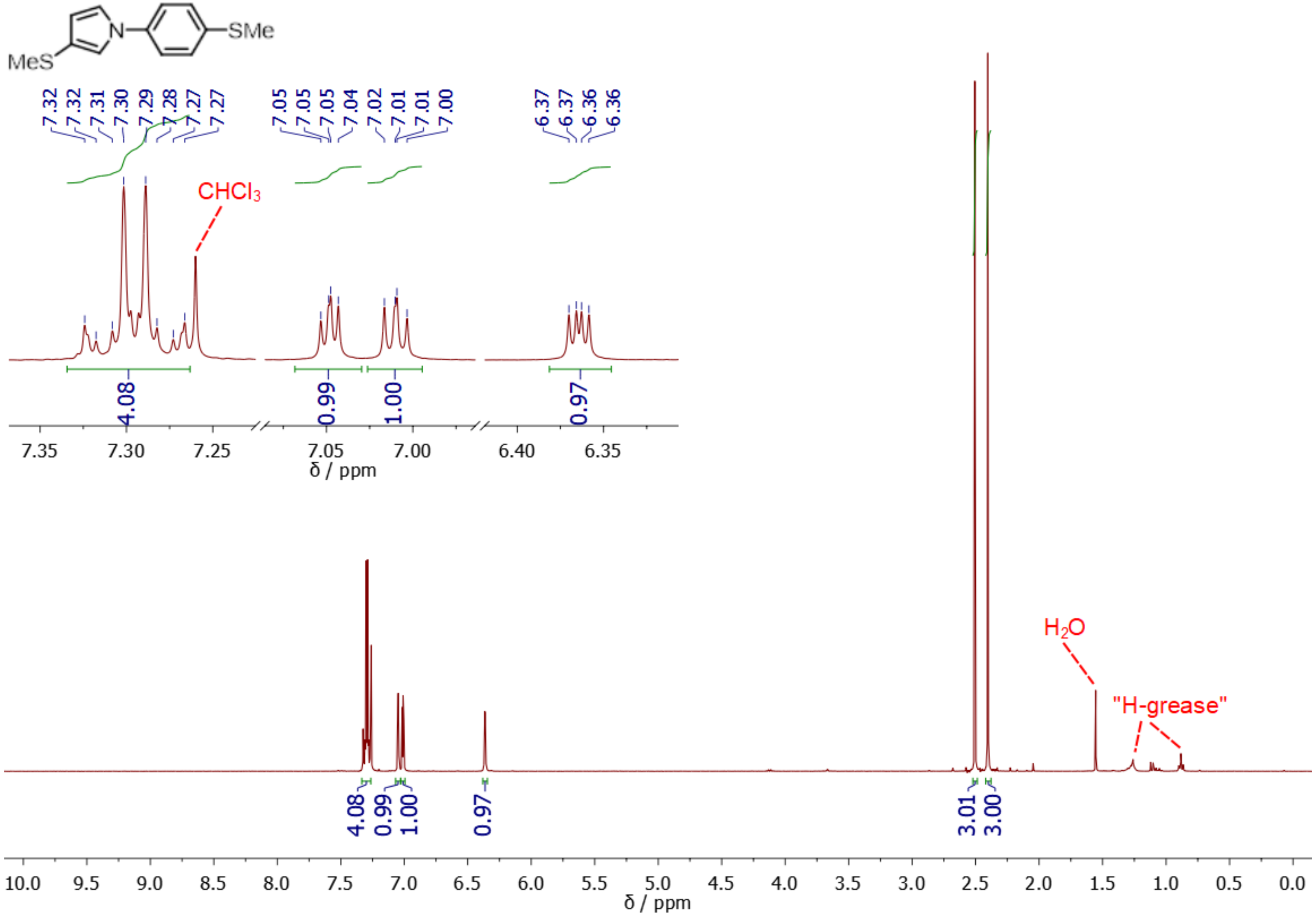

Figure S1. ^{1}H NMR spectrum of compound **1** in $CDCl_3$.

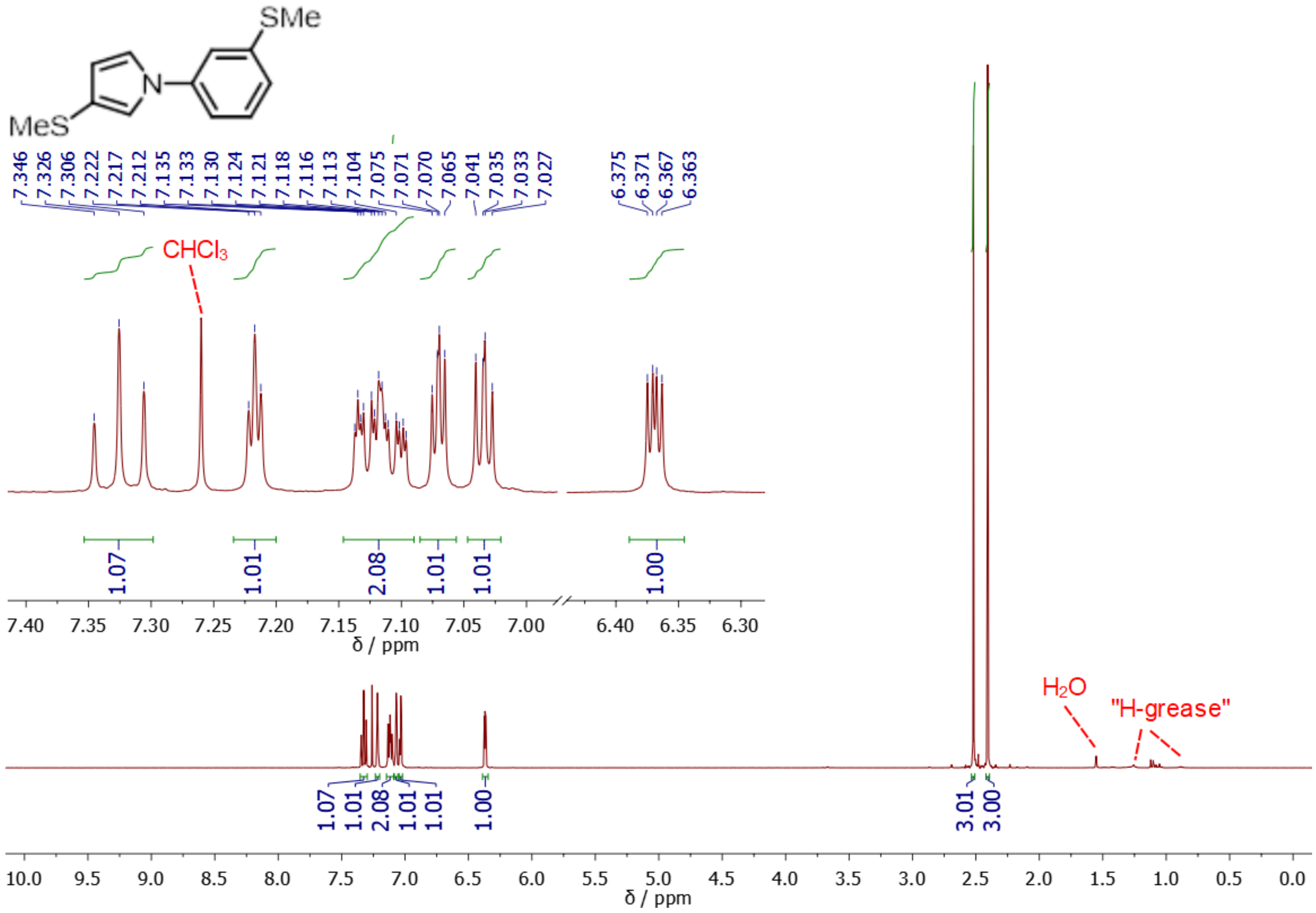

Figure S2. ^{1}H NMR spectrum of compound **2** in $CDCl_3$.

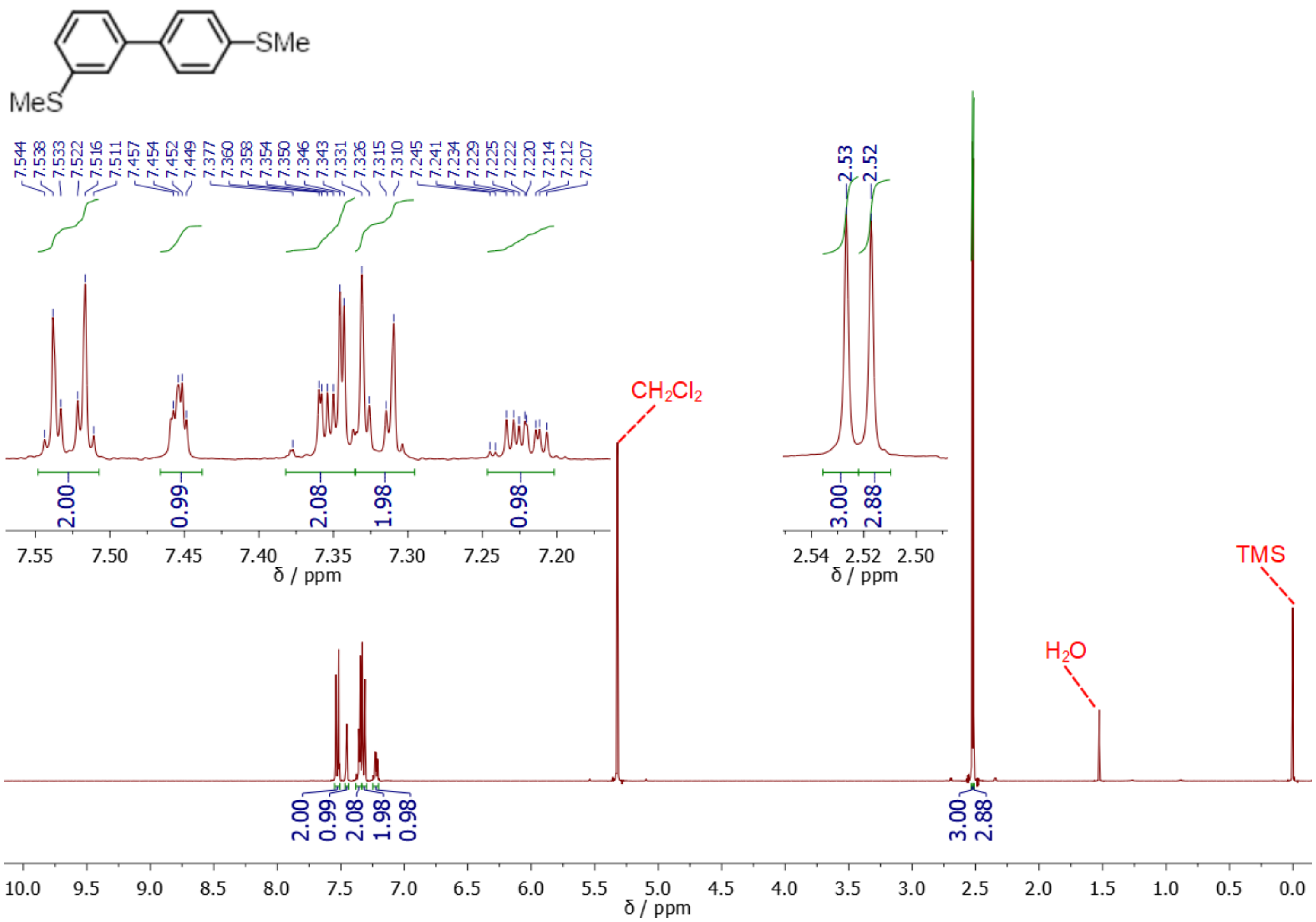

Figure S3. ^{1}H NMR spectrum of compound **3** in CD_2Cl_2.

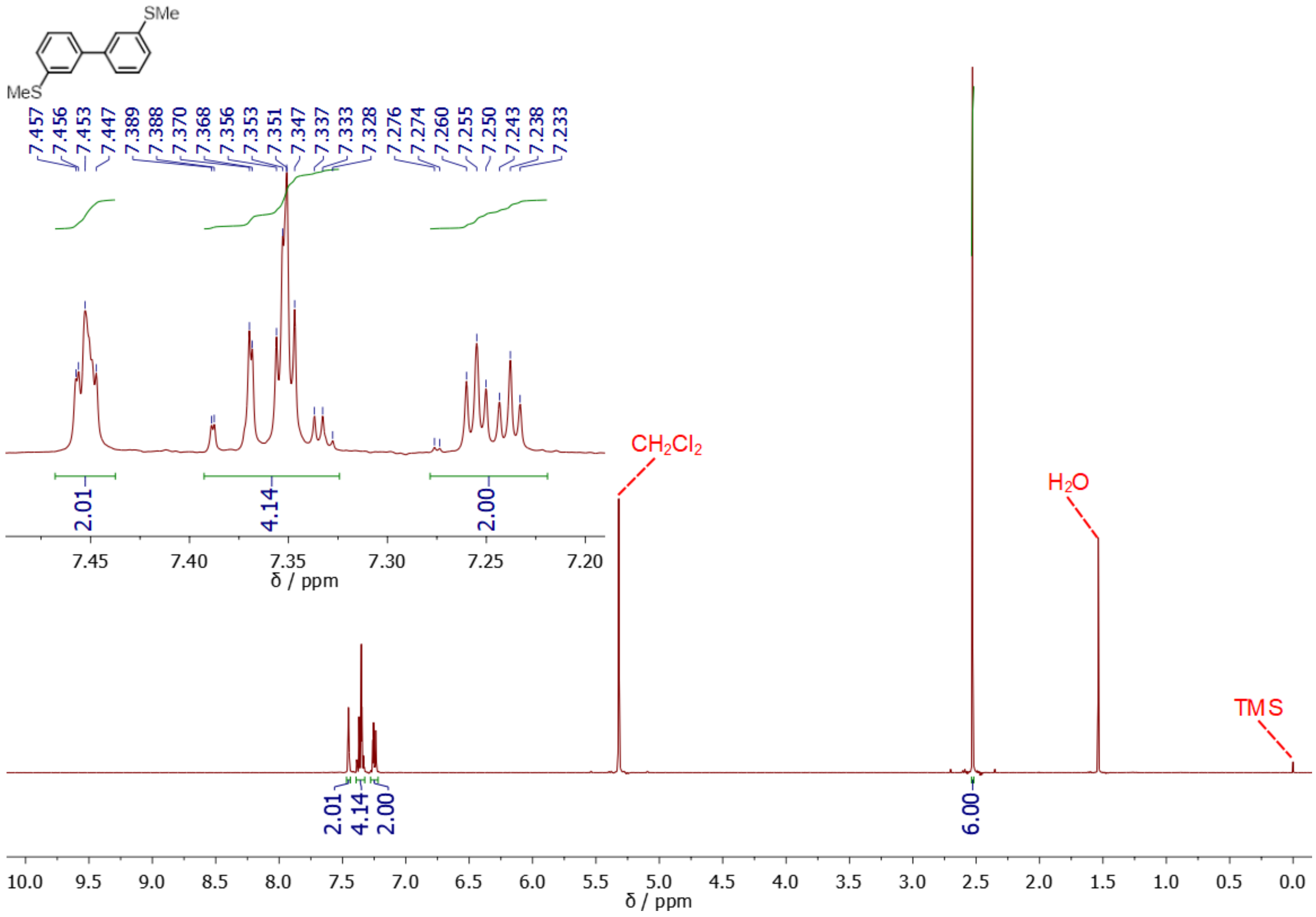

Figure S4. ${}^{1}H$ NMR spectrum of compound **4** in CD_2Cl_2.

1.4: ^{13}C NMR spectra of compounds used in conductance studies

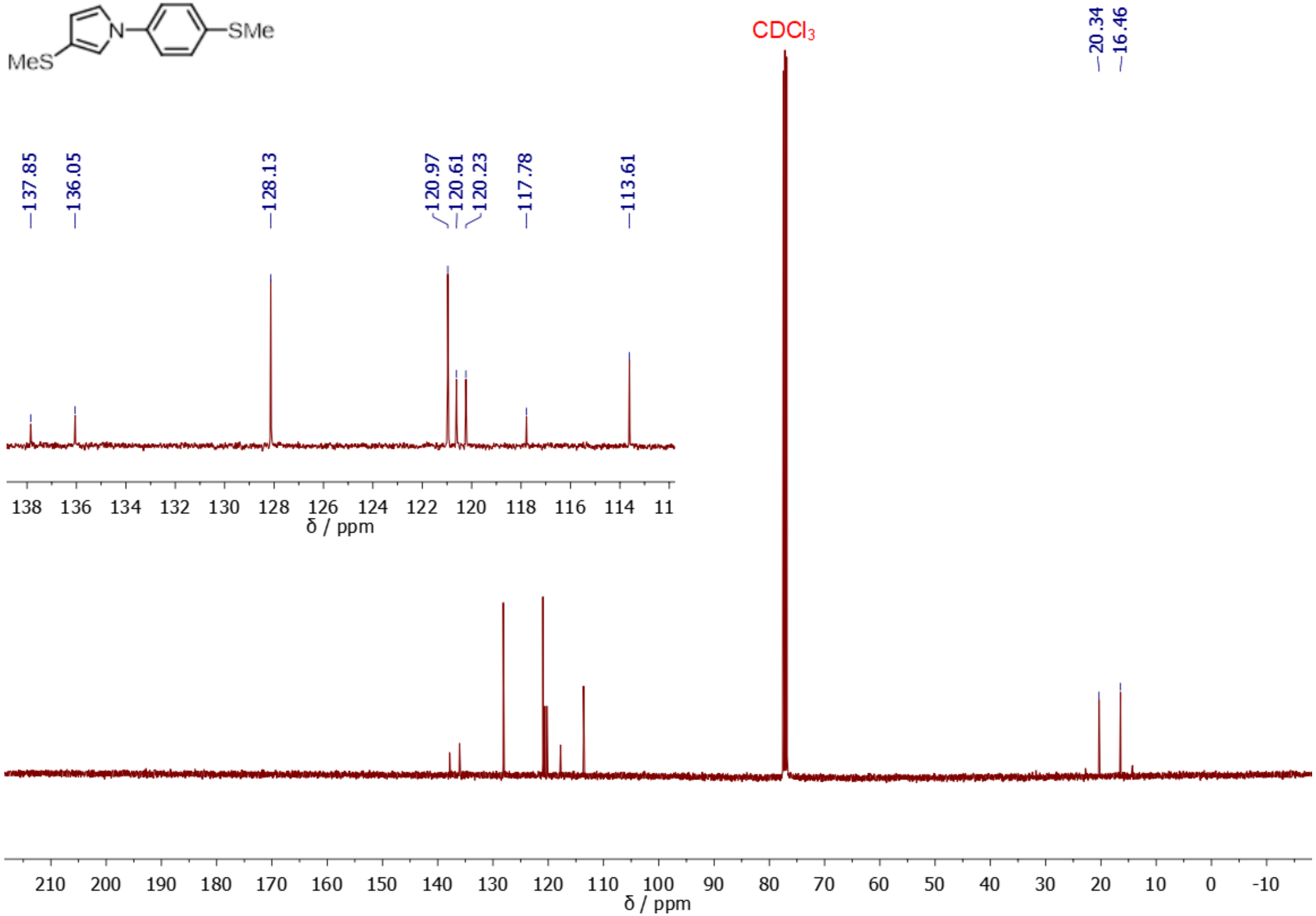

Figure S5. ^{13}C NMR spectrum of compound **1** in $CDCl_3$.

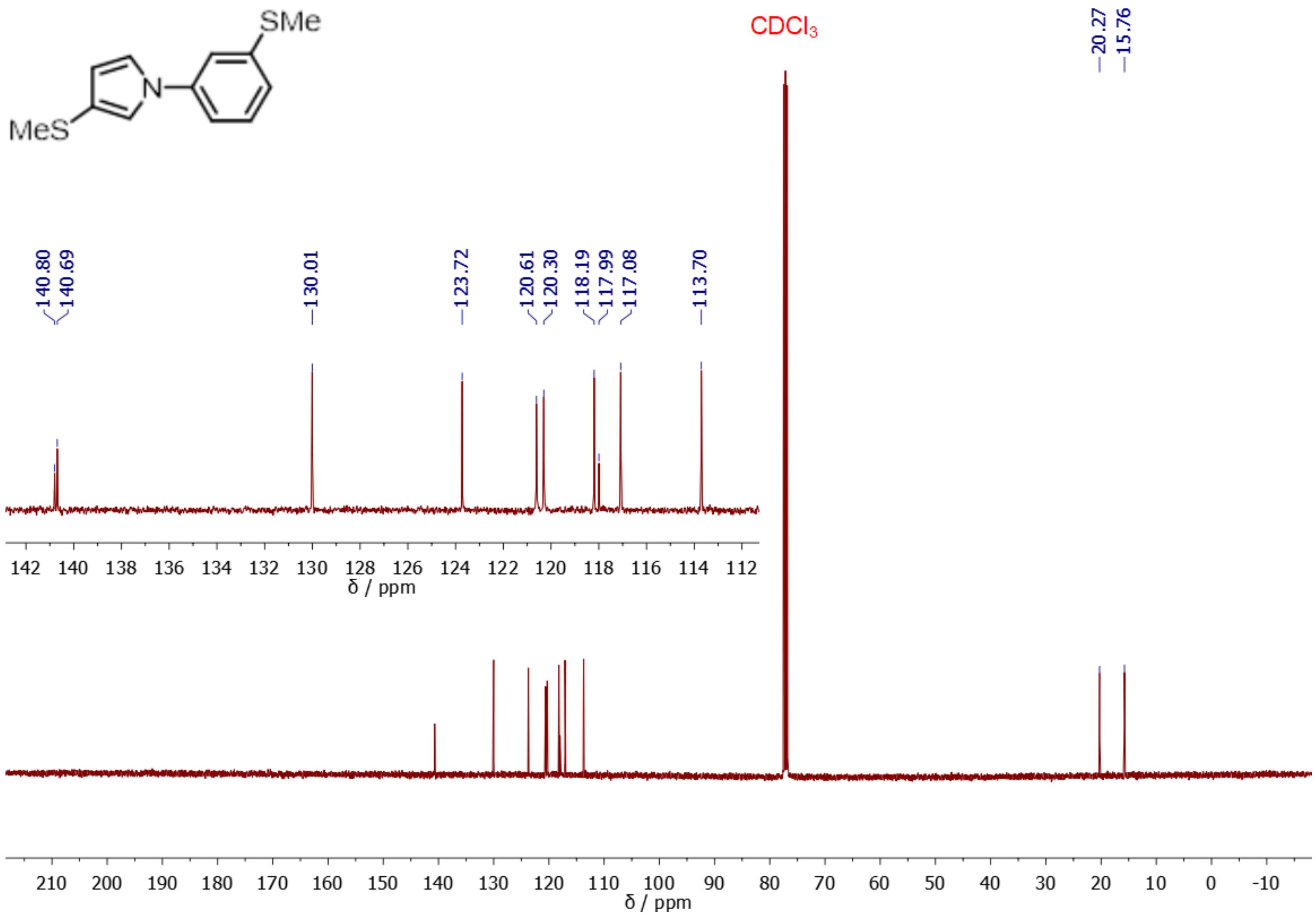

Figure S6. ^{13}C NMR spectrum of compound **2** in $CDCl_3$.

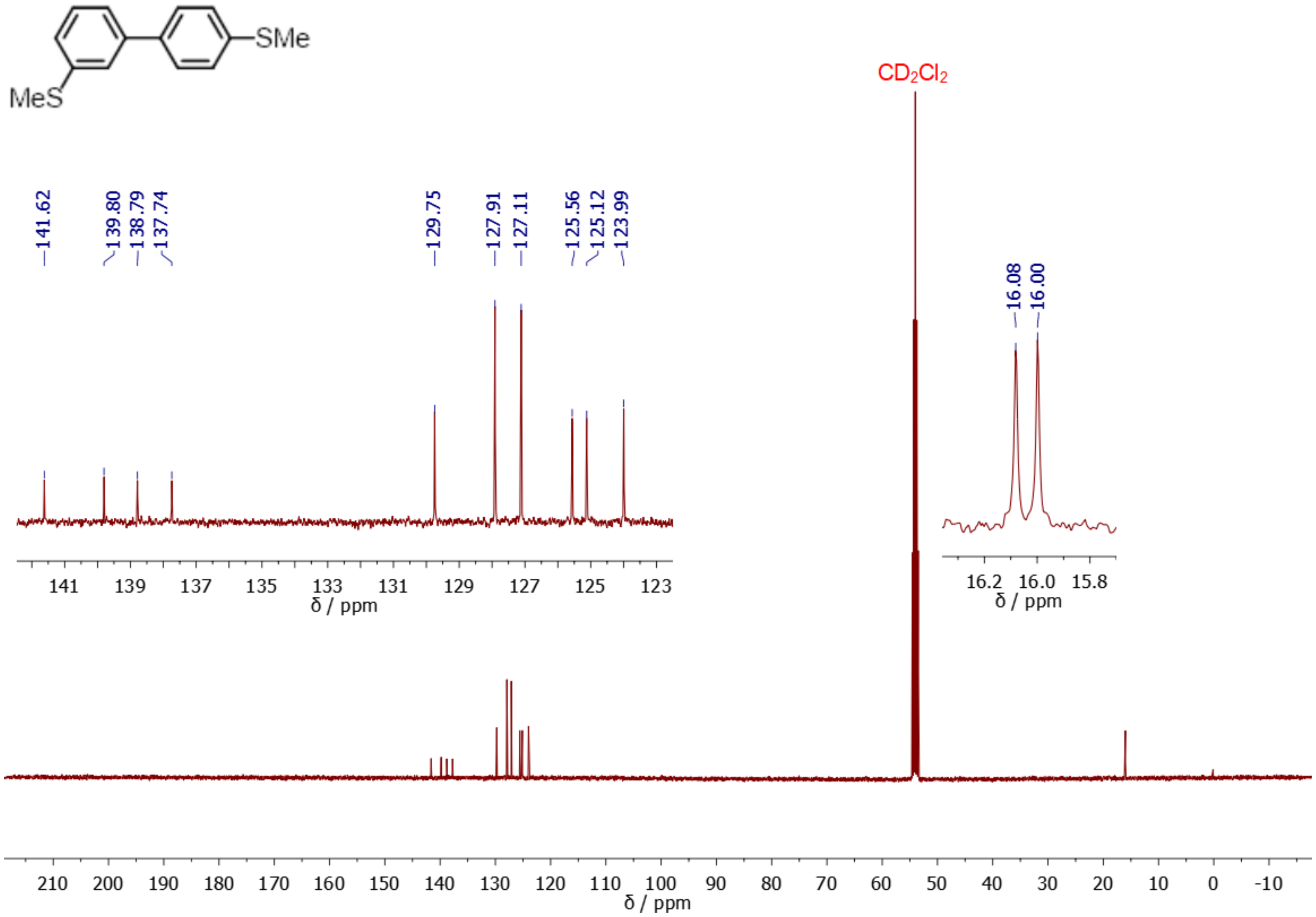

Figure S7. ^{13}C NMR spectrum of compound **3** in CD_2Cl_2.

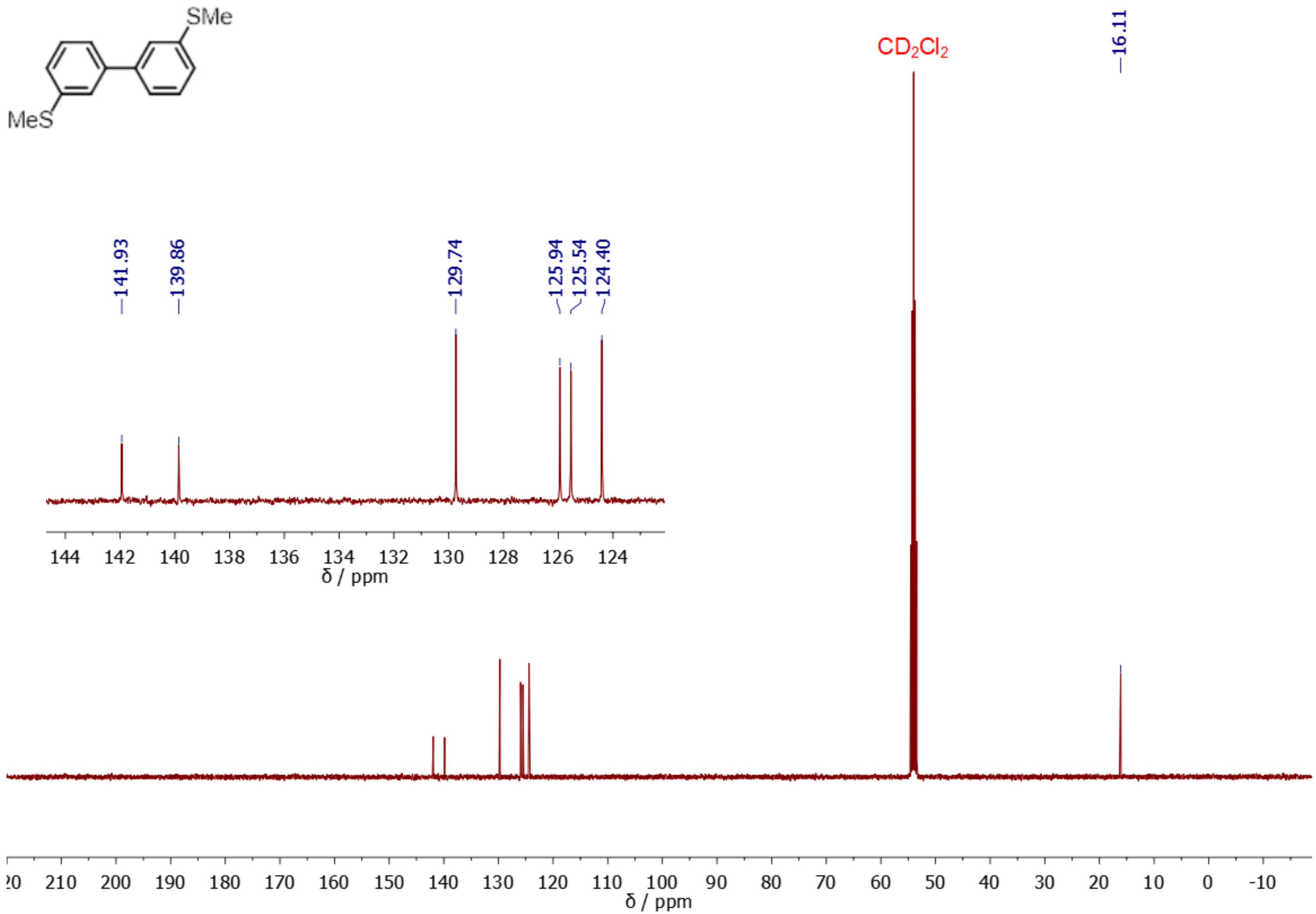

Figure S8. ^{13}C NMR spectrum of compound **4** in CD_2Cl_2.

1.5: UV-visible spectra of molecular wires 1-4

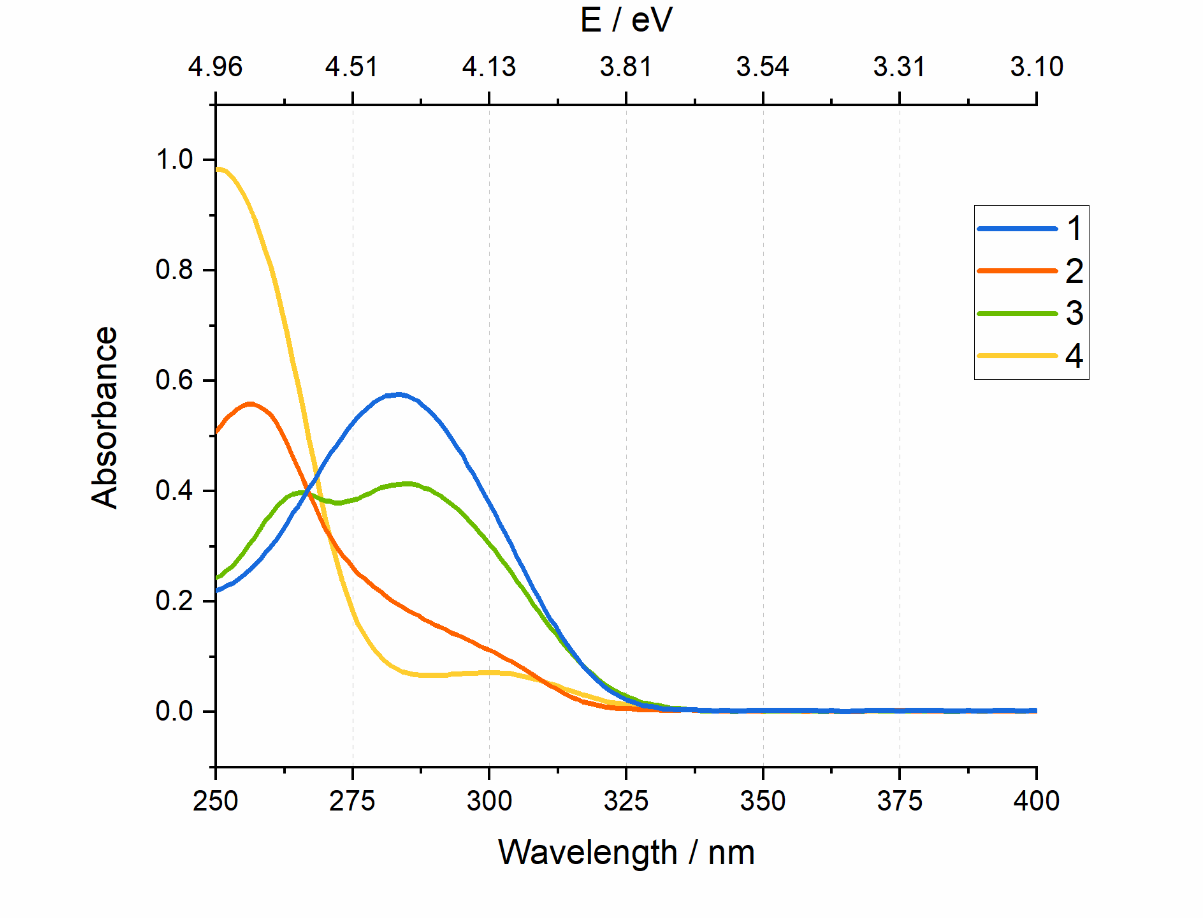

Figure S9. UV-visible spectra of wires **1-4** in CH_2Cl_2 (all ca. 0.02 mM).

2. SINGLE MOLECULE CONDUCTANCE EXPERIMENTS

2.1: Experimental methods

The calculation of the junction length was based on a previously reported method.[8] In brief, the junction displacements were calculated by correcting the piezo stretching rate and tip snapback distance. Firstly, we use the pure mesitylene solvent as a blank experiment and determine the piezo stretching rate by calibrating the direct tunneling distance distribution in mesitylene to the reported value (range from $10^{-3.5}$ G_0 to $10^{-5.5}$ G_0, 0.36 nm). We then use the determined piezo stretching rate to analyze the statistical junction displacements. The final junction displacements were obtained by adding the snapback distance (0.5 ± 0.1 nm).[8]

2.2: Supplementary experimental figures

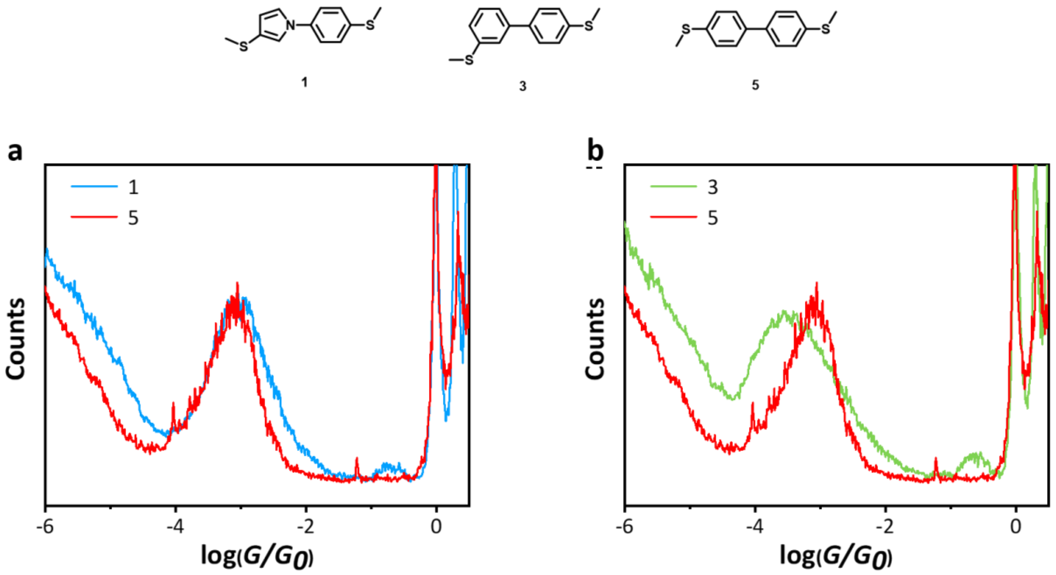

Figure S10. Comparison of logarithmically binned conductance histograms of (a) molecules **1** and **5**,[1] and (b) molecules **3** and **5**, with molecular structures (above).

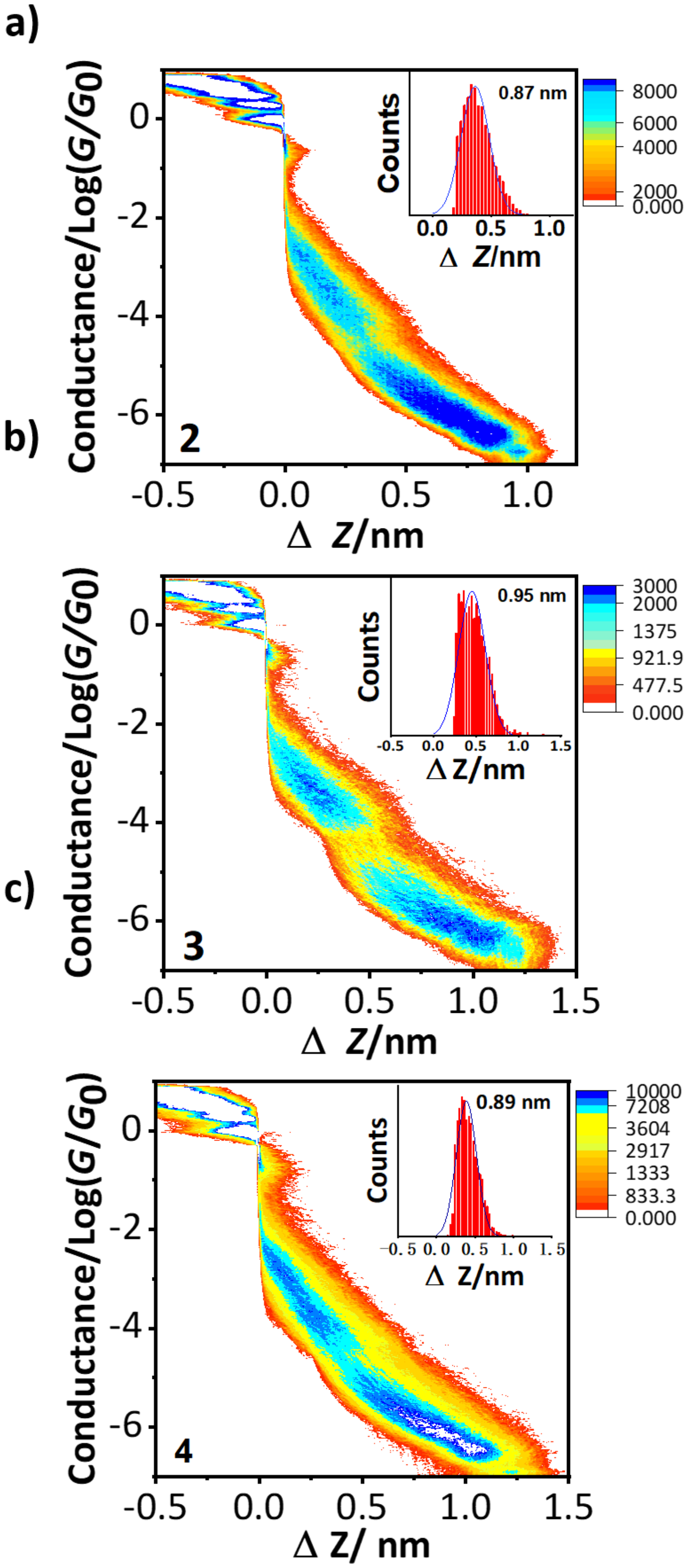

Figure S11. 2D conductance-displacement histograms of molecules (a) **2**, (b) **3** and (c) **4** under 0.1 V bias voltage, insets: length distributions.

3. COMPUTATIONAL STUDIES

3.1: Computational methods

Geometry optimization: The geometry of each molecule studied was relaxed to a force tolerance of 10 meV/Å using the *SIESTA* implementation[9] of density functional theory (DFT), with a double-ζ polarized basis set (DZP) and the Generalized Gradient Approximation (GGA) functional with Perdew-Burke-Ernzerhof (PBE) parameterization. A real-space grid was defined with an equivalent energy cut-off of 250 Ry. To calculate molecular orbitals of gas phase molecules (table 3), we employed experimentally parameterized B3LYP functional using Gaussian g16[10] with 6-311++g basis set and tight convergence criteria.

Electron transport: To calculate electronic properties of the device, from the converged DFT calculation, the underlying mean-field Hamiltonian *H* was combined with the quantum transport code, *Gollum*.[11-12] This yields the transmission coefficient $T(E)$ for electrons of energy E (passing from the source to the drain) via the relation $T(E) = Tr(\Gamma_L\ (E)G^R(E)\Gamma_R\ (E)G^{R\dagger}(E))$ where $\Gamma_{L,R}(E) = i(\sum_{L,R}(E) - {\sum_{L,R}}^{\dagger}(E))$ describes the level broadening due to the coupling between left *L* and right *R* electrodes and the central scattering region, $\Sigma_{L,R}(E)$ are the retarded self-energies associated with this coupling and $G^R = \left(ES - H - \sum_L\ - \sum_R\right)^{-1}$ is the retarded Green's function, where *H* is the Hamiltonian and *S* is the overlap matrix obtained from the *SIESTA* implementation of DFT. The DFT+∑ approach has been employed for spectral adjustment.[12] The electrical conductance is then calculated using the Landauer formula $G(E_F,T) = G_0 \int_{-\infty}^{+\infty} dE\ T(E)(-\partial f(E,T,E_F)/\partial E)$, where $f = (e^{(E-E_F)/k_BT} + 1)^{-1}$ is the Fermi-Dirac probability distribution function, *T* is the temperature, E_F is the Fermi energy, $G_0 = 2e^2/h$ is the conductance quantum, *e* is electron charge and *h* is the Planck's constant.

3.2: Supplementary computational figures, tables and discussion

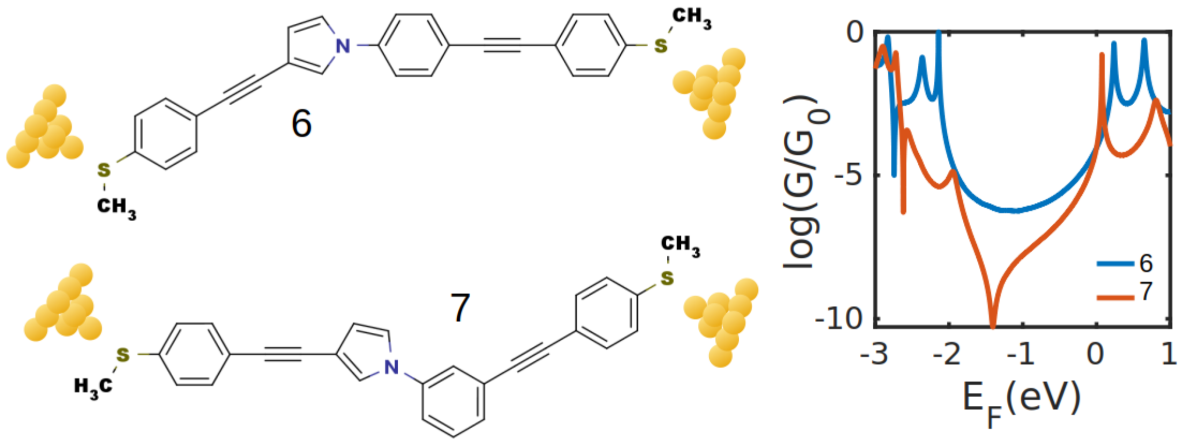

Figure S12. Left: Illustration of molecules **6** and **7**, extended analogs of molecules **1** and **2**, in molecular junctions. Right: Calculated electron transport through molecules **6** and **7** between gold electrodes using DFT material-specific Hamiltonians.

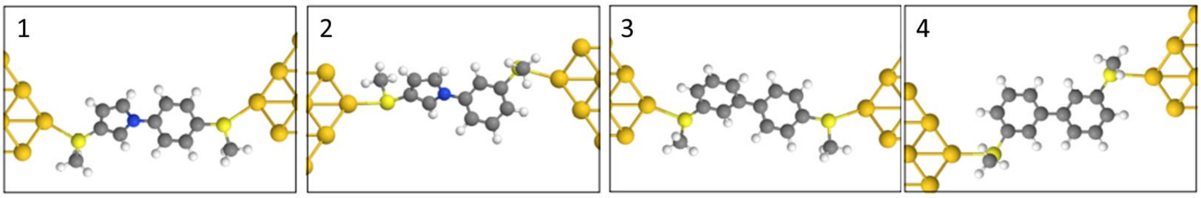

Figure S13. DFT-optimized configurations of molecules **1**-**4** between two gold electrodes.

Table S1. Torsion angle (θ) between aromatic rings of molecular wires **1**-**4** in the DFT-optimized junction geometry between two gold electrodes and in gas phase.

Molecule	Aryl-aryl torsion angle (θ) in gas phase molecules	Aryl-aryl torsion angle (θ) in junctions between gold electrodes
**1**	38.1°	43.3°
**2**	37.9°	37.5°
**3**	39.8°	34.8°
**4**	39.6°	27.2°

Table S2. Calculated molecular conductances at E_F = 0 eV from charge transport simulations using DFT material-specific Hamiltonians

Molecule	Conductance at $E_F = 0$ *eV* / ($\log(G/G_0)$)
**1**	-3.81
**2**	-4.38
**3**	-4.26
**4**	-4.50

Table S3. Molecular orbitals of molecules **1**-**4**.

	HOMO-2	HOMO-1	HOMO	LUMO	LUMO+1
**1**	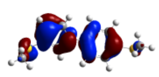	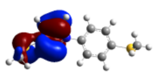	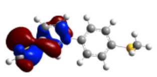	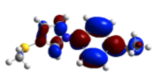	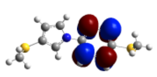
**2**	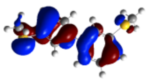	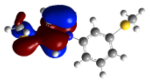	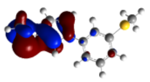	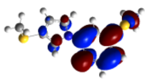	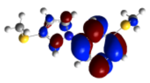
**3**	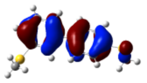	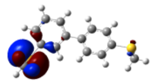	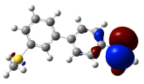	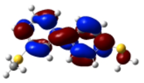	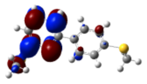
**4**	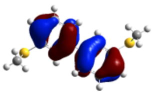	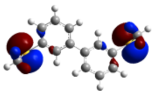	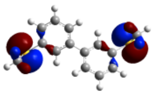	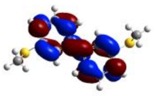	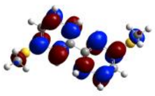

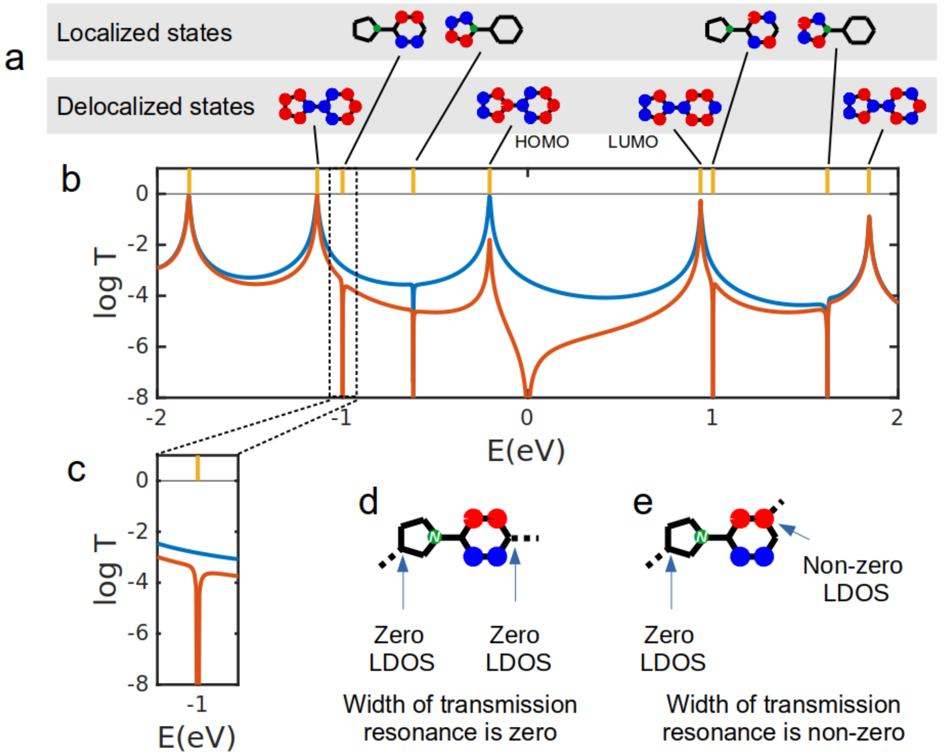

Figure S14. Tight-binding orbitals and transmissions for **1** and **2**. (a) The molecular orbitals (wave functions) corresponding to energy levels of gas phase molecules marked by orange lines in panel b. (b) Tight-binding transmission functions for **1** and **2**. (c) Expansion of the energy range shown by dashed line in panel b. Tight-binding molecular orbitals for the energy level around $E = -1$ eV, showing that the local density of state (LDOS) is zero at both connection points of **1** (d), but non-zero at one connection point of **2** (e).

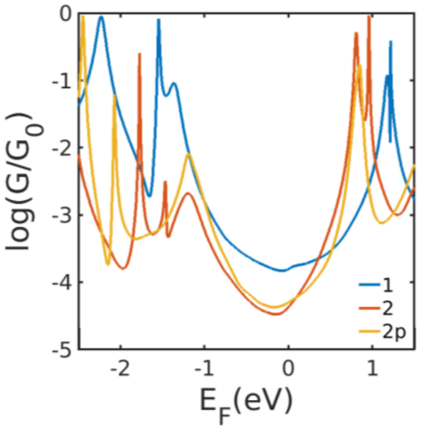

Figure S15. Transmission coefficients for **1** and two configurations of **2** using DFT material-specific Hamiltonians. The blue and red curves are those shown in Figure 3a in the main text and correspond to the ground state geometry of the junctions. The orange curve corresponds to an alternative conformation of **2** in which the dihedral angle Au-S-C-C is set to be the same as that of ground state junction geometry of **1**.

The differing junction geometries of **1** and **2** (Figure S13) mean that the dihedral angle Au-S-C-C at the anchoring point is different in each case. It is known that this angle can influence the electrical conductance of molecular wires.[13] To rule out the possibility that the relative conductance of the two isomers was significantly affected by this dihedral angle, an alternative conformation of **2** in a junction ("**2p**") was investigated, in which the dihedral angle Au-S-C-C was constrained to be the same as that of **1** in its ground state junction conformation. These calculations show that this new junction conformation is energetically ca. 1.25 eV less favorable than the ground state geometry. The resulting DFT-based transmission function (Figure S15) for this new **2p** configuration shows no significant difference in the HOMO-LUMO gap compared to the ground state

conformation of **2**. It can be concluded that the Au-S-C-C dihedral angle does not have significant influence on the discussed conductance trends or conductance ratios between isomers.

4. REFERENCES

(1) Tang, Y.; Zhou, Y.; Zhou, D.; Chen, Y.; Xiao, Z.; Shi, J.; Liu, J.; Hong, W., Electric Field-Induced Assembly in Single-Stacking Terphenyl Junctions. *J. Am. Chem. Soc.* **2020,** *142*, 19101-19109.

(2) Gottlieb, H. E.; Kotlyar, V.; Nudelman, A., NMR Chemical Shifts of Common Laboratory Solvents as Trace Impurities. *J. Org. Chem.* **1997,** *62*, 7512-7515.

(3) John, J.; Gravel, E.; Hagège, A.; Li, H.; Gacoin, T.; Doris, E., Catalytic Oxidation of Silanes by Carbon Nanotube–Gold Nanohybrids. *Angew. Chem. Int. Ed.* **2011,** *50*, 7533-7536.

(4) O'Driscoll, L. J.; Hamill, J. M.; Grace, I.; Nielsen, B. W.; Almutib, E.; Fu, Y.; Hong, W.; Lambert, C. J.; Jeppesen, J. O., Electrochemical control of the single molecule conductance of a conjugated bis(pyrrolo)tetrathiafulvalene based molecular switch. *Chem. Sci.* **2017,** *8*, 6123-6130.

(5) Antilla, J. C.; Baskin, J. M.; Barder, T. E.; Buchwald, S. L., Copper−Diamine-Catalyzed N-Arylation of Pyrroles, Pyrazoles, Indazoles, Imidazoles, and Triazoles. *J. Org. Chem.* **2004,** *69*, 5578-5587.

(6) Klausen, R. S.; Widawsky, J. R.; Su, T. A.; Li, H.; Chen, Q.; Steigerwald, M. L.; Venkataraman, L.; Nuckolls, C., Evaluating atomic components in fluorene wires. *Chem. Sci.* **2014,** *5*, 1561-1564.

(7) Zweig, A.; Maurer, A. H.; Roberts, B. G., Oxidation, reduction, and electrochemiluminescence of donor-substituted polycyclic aromatic hydrocarbons. *J. Org. Chem.* **1967,** *32*, 1322-1329.

(8) Hong, W.; Manrique, D. Z.; Moreno-García, P.; Gulcur, M.; Mishchenko, A.; Lambert, C. J.; Bryce, M. R.; Wandlowski, T., Single Molecular Conductance of Tolanes: Experimental and Theoretical Study on the Junction Evolution Dependent on the Anchoring Group. *J. Am. Chem. Soc.* **2012,** *134*, 2292-2304.

(9) Soler, J. M.; Artacho, E.; Gale, J. D.; García, A.; Junquera, J.; Ordejón, P.; Sánchez-Portal, D., The SIESTA method for ab initio order- N materials simulation. *J. Phys.: Condens. Matter* **2002,** *14*, 2745.

(10) Frisch, M. J.; Trucks, G. W.; Schlegel, H. B.; Scuseria, G. E.; Robb, M. A.; Cheeseman, J. R.; Scalmani, G.; Barone, V.; Petersson, G. A.; Nakatsuji, H., et al. *Gaussian 16 Rev. C.01*, Wallingford, CT, 2016.

(11) Ferrer, J.; Lambert, C. J.; García-Suárez, V. M.; Manrique, D. Z.; Visontai, D.; Oroszlany, L.; Rodríguez-Ferradás, R.; Grace, I.; Bailey, S. W. D.; Gillemot, K., et al. GOLLUM: a next-generation simulation tool for electron, thermal and spin transport. *New J. Phys.* **2014,** *16*, 093029.

(12) Sadeghi, H., Theory of electron, phonon and spin transport in nanoscale quantum devices. *Nanotechnology* **2018,** *29*, 373001.

(13) Park, Y. S.; Widawsky, J. R.; Kamenetska, M.; Steigerwald, M. L.; Hybertsen, M. S.; Nuckolls, C.; Venkataraman, L., Frustrated Rotations in Single-Molecule Junctions. *J. Am. Chem. Soc.* **2009,** *131*, 10820-10821.
